# Immunological landscape of consensus clusters in colorectal cancer

**DOI:** 10.18632/oncotarget.22169

**Published:** 2017-10-27

**Authors:** Pawel Karpinski, Joanna Rossowska, Maria Malgorzata Sasiadek

**Affiliations:** ^1^ Department of Genetics, Wroclaw Medical University, Wroclaw, Poland; ^2^ L. Hirszfeld Institute of Immunology and Experimental Therapy, Polish Academy of Sciences, Wroclaw, Poland

**Keywords:** colorectal, clusters, immunotherapy, immune modifiers

## Abstract

Recent, large-scale expression–based subtyping has advanced our understanding of the genomic landscape of colorectal cancer (CRC) and resulted in a consensus molecular classification that enables the categorization of most CRC tumors into one of four consensus molecular subtypes (CMS). Currently, major progress in characterization of immune landscape of tumor-associated microenvironment has been made especially with respect to microsatellite status of CRCs. While these studies profoundly improved the understanding of molecular and immunological profile of CRCs heterogeneity less is known about repertoire of the tumor infiltrating immune cells of each CMS.

In order to comprehensively characterize the immune landscape of CRC we re-analyzed a total of 15 CRC genome-wide expression data sets encompassing 1597 tumors and 125 normal adjacent colon tissues. After quality filtering, CRC clusters were discovered using a combination of multiple clustering algorithms and multiple validity metrics. CIBERSORT algorithm was used to compute relative proportions of 22 human leukocyte subpopulations across CRC clusters and normal colon tissue. Subsequently, differential expression specific to tumor epithelial cells was calculated to characterize mechanisms of tumor escape from immune surveillance occurring in particular CRC clusters.

Our results not only characterize the common and cluster-specific influx of immune cells into CRCs but also identify several deregulated gene targets that may contribute to improvement of immunotherapeutic strategies in CRC.

## INTRODUCTION

The Cancer Genome Atlas (TCGA) and other large-scale cancer molecular profiling efforts showed that colorectal cancer (CRC) is a heterogeneous disease, arising from a number of possible etiological pathways that are responsible for driving CRC development [1]. Recent integration of various CRC gene expression-based subtypings resulted in a consensus molecular CRC classification that enables the segregation of most tumors into one of four consensus molecular subtypes (CMS) [2]. Each of CRC CMS has been marked by distinct driver mutations and genetic or epigenetic signatures that include microsatellite instability (MSI), CpG island methylator phenotype (CIMP), chromosomal instability (CIN) and diverse spectrum of pathway activation (for details see Dienstmann *et al*. [3]). A gene-set enrichment analysis of gene expression data has provided initial insight into immune microenvironment CMS groups [3, 4]. It has been demonstrated that CMS1 tumors are characterized by high infiltration of immune cells associated with adaptive immunity. In contrast, CMS4 displays so called “inflamed phenotype” associated with expansion of innate immunity cells and expression of immunosuppressive factors [3]. Finally, CMS2 and CMS3 exhibit low immune system activation.

Currently, we are witness to major progress in detailed characterization of immune landscape of tumor microenvironment, especially with respect to microsatellite status (microsatellite-stable [MSS] and microsatellite-instable [MSI]) of CRCs [5]. Recent high –resolution genomic and transcriptomic analyses of CRC have uncovered a strong positive correlation between high mutational load, lymphocytic infiltration and prolonged patient survival [5, 6]. Lately, Angelova *et al*. and Giannakis [6] *et al*. performed detailed lymphocyte profiling of CRCs classified by MSI/MSS status and tumor mutational heterogeneity [5, 6]. Both studies showed that MSI tumors have favorable immune (increased influx of cytotoxic T lymphocytes) and genetic characteristics (high neo-antigen load) when compared to MSS tumors (reduced levels of cytotoxic T cells, low mutational load, presence of immunosuppressive cells). Finally, above mentioned studies discussed the mechanisms of tumor escape from immune surveillance for example associated with upregulation of immunoinhibitory molecules (PD-1), positive selection of HLA mutations or downregulation of major histocompatibility complex (MHC) molecules [5]. While these studies profoundly improved the understanding of molecular and immunological profile of CRCs heterogeneity less is known about the immune landscape (that is, the repertoire of the tumor infiltrating immune cells) of each consensus molecular CRC subtype.

In the present study, we performed robust expression subtype classification of CRCs followed by computation of relative proportions of 22 subpopulations of human leukocytes across 4 identified CRC transcriptional subtypes and normal colon tissues. We provided detailed repertoire of the tumor infiltrating immune cells for each CRC subtype. Finally, we identified significantly deregulated immune-related genes in epithelial tumor cells that may significantly influence the activity of immune cells infiltrating different CRC clusters.

## RESULTS

### Identification of transcriptional subtypes in CRC and normal tissue content estimations

**Figure 1:**
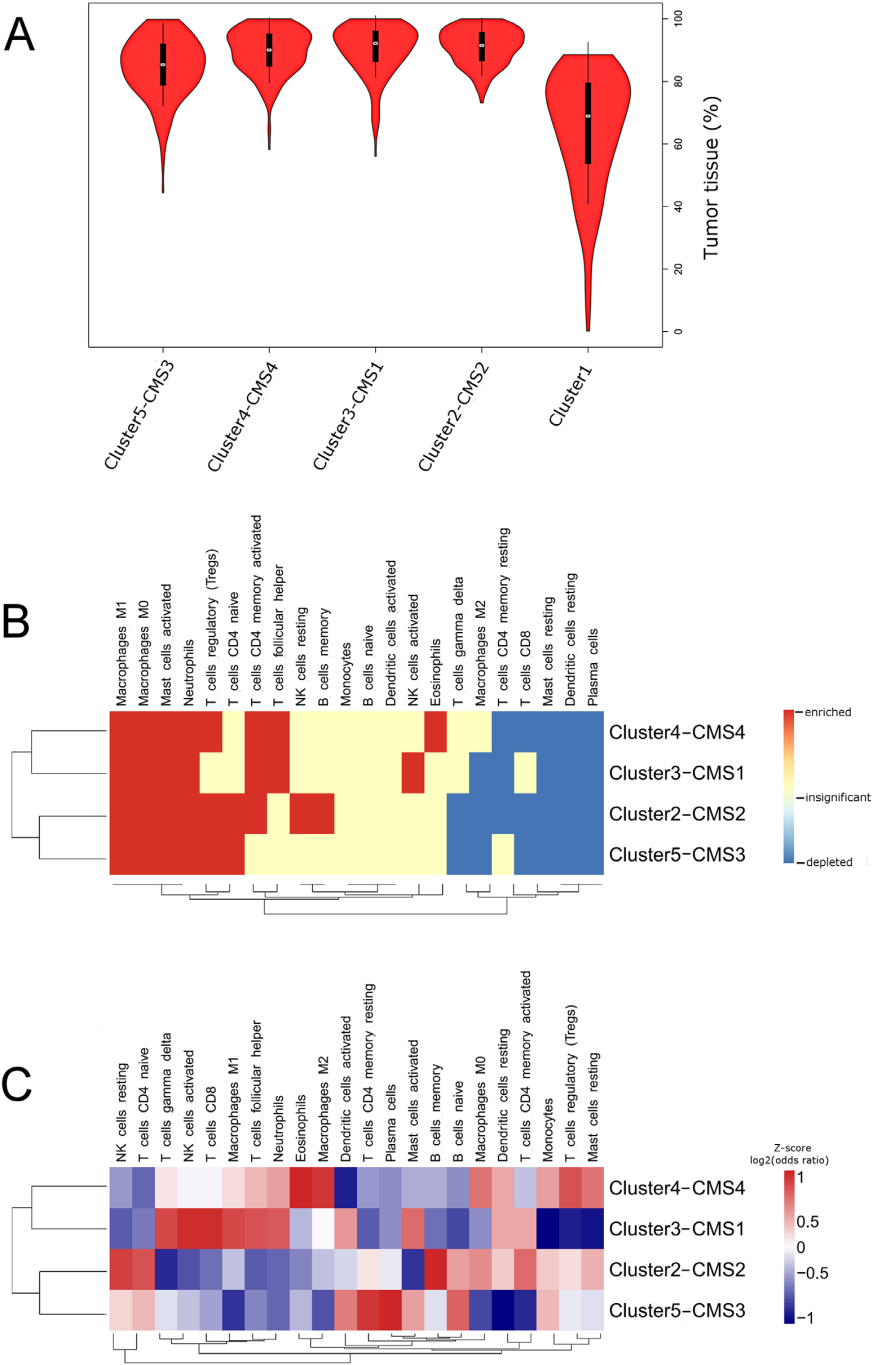
**(A)** Violin plots illustrating densities of tumor purities in five COMMUNAL clusters computed by “ISOpureR” package. Median is marked with a white circle. Note the significantly lower tumor purities in Cluster1 (cluster with the highest normal tissue content). **(B and C)** Clustered heatmaps (Euclidean distance, average linkage) characterizing leukocyte subpopulations in CRC subtypes. (B) Comparison between normal colon and CRC subtypes. Enrichment (red) has been defined as positive mean fold-change and adjusted p-value ≤ 0.05. Depletion (blue) has been defined as negative mean fold-change and adjusted p-value ≤ 0.05. Insignificant differences (white) had adjusted p-value ≥ 0.05.(C) Comparison between CRC subtypes presented as a log-transformed odd ratios provided by CRC subtype *versus* remaining samples comparison. For better visualization, rows were scaled to z-score after calculating the dendogram.

In the present study, we initially performed analysis of RNA degradation measurements across 15 CRC genome-wide expression data sets that included 1597 tumor and 125 adjacent normal colon samples. After excluding samples with high RNA degradation (d^k^ < 0.45), we conducted unsupervised clustering on a group of 1492 CRCs using COMMUNAL algorithm [7, 8]. COMMUNAL incorporates information from multiple variable subsets, clustering algorithms and validity metrics, therefore it allows a reliable identification of transcriptional subtypes. Using this approach K=5 stable CRC clusters were revealed (Supplementary Figure 1). We then computed normal tissue content for each sample in each COMMUNAL cluster using gene expression profiles of normal colon samples as a reference [9]. Comparison of tumor purity between clusters revealed that Cluster 1 (composed of 226 samples) had significantly lower tumor purity (mean = 0.65) when compared to the other clusters (adjusted p –value < 0.001 for all comparisons, data not shown) (Figure 1A). Given that the presence of a high proportion of normal cells in the sample may significantly affect all other downstream computations we decided to remove samples that were included in Cluster 1. Therefore all subsequent analyses were performed on the remaining 1225 CRCs.

We next compared our cluster assignments to consensus clustering subtypes (CMS) published by Guinney *et al*. based on 896 samples that were included in our study [2]. As shown in Table 1 we found statistically significant overlap between cluster2 and CMS2 (92 % samples), cluster3 and CMS1 (87 % samples), cluster4 and CMS4 (87 % samples), cluster5 and CMS3 (71 % samples). Given, that CMS division of CRCs is highly recognized in recent literature we decided to combine COMMUNAL cluster labels with that proposed by Guinney *et al*. (Cluster2-CMS2, Cluster3-CMS1, Cluster4–CMS4 and Cluster5-CMS3).

**Table 1:** Molecular characteristics of 1225 CRCs (after quality filtering and exclusion of samples from Cluster1)

	All samples	Cluster2	Cluster3	Cluster4	Cluster5
	n=1225	n=456	n=250	n=296	n=223
**MSI**	237 (19.3%)	5 (1.1%)	**180 (72.0%)**	17 (5.7%)	35 (15.7%)
**MSS**	988 (80.7%)	451 (98.9%)	70 (28.0%)	279 (94.3%)	188 (84.3%)
**Age**	69.0 [60.0;77.0]	70.0 [61.5;76.5]	72.0 [63.5;78.8]	67.0 [57.2;75.0]	69.0 [60.0;76.8]
**female**	261 (44.0%)	91 (40.8%)	66 (61.7%)	55 (37.9%)	49 (41.5%)
**male**	332 (56.0%)	132 (59.2%)	41 (38.3%)	90 (62.1%)	69 (58.5%)
**CMS1**	173 (19.3%)	1 (0.3%)	**156 (87.2%)**	2 (1.0%)	14 (8.0%)
**CMS2**	343 (38.3%)	**312 (92.0%)**	0 (0.0%)	9 (4.4%)	22 (12.6%)
**CMS3**	126 (14.1%)	0 (0.0%)	2 (1.1%)	0 (0.0%)	**124 (71.3%)**
**CMS4**	201 (22.4%)	10 (2.9%)	13 (7.3%)	**178 (87.3%)**	0 (0.0%)
**NOLBL**	53 (5.9%)	16 (4.7%)	8 (4.5%)	15 (7.4%)	14 (8.0%)

Microsatellite instability data (MSI/MSS) includes 671 CRC samples with known MSI status and 554 CRC samples with predicted MSI status. Age, gender and CMS cluster variables were available for 473 samples, 593 samples and 896 samples, respectively. ‘NOLBL’ stands for samples that could not be attributed to any CMS subtype (for details see main text, ref. Guinney *et al*. 2015).

### Microsatellite instability classifier

Given that MSI status in one of the most important molecular correlates in studies related to immune-cell infiltrates in CRC we developed binary MSI classifier to predict microsatellite status in 554 out of 1225 samples lacking this information. Classifier average sensitivity and recall calculated over random 20 test sets were 0.88 and 0.9 for the MSI class and 0.97 and 0.96 for the MSS class, respectively. Supplementary Figure 2 provides visualization of performance of MSI classifier acrossrandom 20 test sets.

After imputation of the missing MSI status we found out that 19% of tumors were classified as MSI. As shown in Table 1, Cluster3-CMS1 was significantly enriched with MSI tumors (72% samples, adjusted p-value < 0.001 for all pairwise comparisons) when compared to other clusters. Cluster5-CMS3 displayed moderate enrichment with MSI tumors (16% samples) whereas in Cluster2-CMS2 and Cluster4-CMS4 MSI tumors were rare (1% and 6% samples, respectively). All associations between COMMUNAL clusters and MSI status are in agreement with those presented by Guinney *et al*. for CMS [3].

### Differences in tissue infiltrating leukocyte proportions between CRC subtypes and normal colon tissue

Using the leukocyte gene signature (LM22) and CIBERSORT algorithm we computed relative proportions of 22 leukocyte subpopulations in 1225 tumor and 83 normal colon samples. Detailed characteristics of cells included in LM22 and abbreviations we used are available in Supplementary Table 1. Next we used computed proportions to characterize profile of leukocyte influx of each CRC subtype and normal colon. Violin plots visualize the densities of computed proportions of each leukocyte subpopulation in each CRC cluster and normal colon (Supplementary Figure 3). We used clustered heatmap to visualize immune cells that were enriched, depleted or did not show statistically significant changes when compared to normal colon (Figure 1B). In Supplementary Figure 4 we provided volcano plots that visualize average fold change in addition to statistical significance for CRC subtype *versus* normal colon comparisons.

In comparison to normal colon tissue for all 4 CRC clusters we observed significant enrichment of innate immune cells (macrophages M0 and M1, activated mast cells and neutrophils). In contrast, plasma cells, resting mast cells and resting DCs displayed significant depletion in all CRC groups. Frequent enrichment (for 3 out of 4 clusters) was also observed for: Tregs (except Cluster3–CMS1) and memory activated Th (except Cluster5-CMS3)), whereas, frequent depletion was observed in respect to CTLs (except Cluster3–CMS1), memory resting Th (except Cluster5-CMS3), and macrophages M2 (except Cluster4-CMS4).

### Comparison of tumor infiltrating leukocyte proportions between CRC subtypes

To enable visualization of subtle differences in the immune cell influx between CRC subtypes we clustered log-transformed odd ratios provided by comparing CRC subtype *versus* remaining CRCs (Figure 1C; see alsoviolin plots provided in Supplementary Figure 3). Cluster3–CMS1 displayed both, enrichment of leukocytes related to adaptive immunity (Tfh, memory activated Th and CTLs) and to innate immunity (activated NK cells, γδT cells, M1 macrophages, activated DCs, activated mast cells and neutrophils) [10, 11]. In addition, Cluster3–CMS1 was characterized by depletion of Tregs. Cluster4–CMS4 was characterized by highest proportions of leukocytes related to pro-tumor activity (eosinophils, monocytes, macrophages M2, resting DCs and Tregs) and depletion of activated DCs. Cluster2-CMS2 displayed the highest enrichment in Th cells (naive and memory activated) and memory B cells. Finally, the Cluster5-CMS3 displayed relatively low levels of immune activation that was manifested by high levels of memory resting Th, naive B cells and low levels of macrophages, neutrophils and activated Th.

### Characterization of tumor cellular composition and immune escape mechanisms in CRC clusters

**Figure 2:**
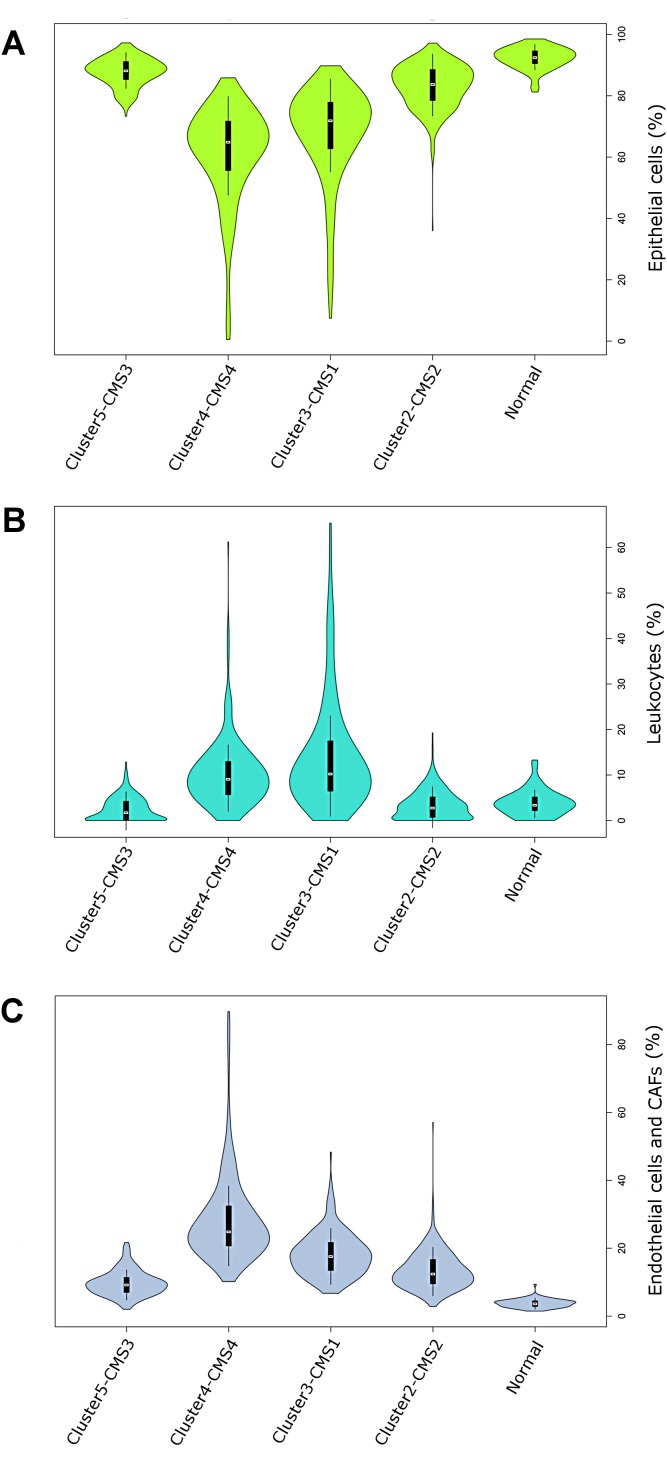
**(A-C)** Violin plots visualizing the densities of computed proportions of 3 main tumor compartments: epithelial cells (A), leukocytes (B) endothelial cells and CAFs (Epcam (−) / CD45 (−)) (C) in each CRC cluster and normal colon samples. Mean is marked with a white circle.

To identify genes potentially involved in tumor-specific immune response regulation, we determined the differences in immune-related gene expression in tumor epithelial cells for each CRC subtype. This required construction of expression signature specific for cell types that form tumor (epithelial cells, leukocytes, endothelial cells and cancer associated fibroblasts (CAFs)), calculation of relative cell proportions and subsequent calculation of differential expression specific to each cell fraction. Available data enabled us to construct expression signature for epithelial cells, leukocytes and endothelial cells combined with CAFs. Estimated relative proportions of each tumor compartment are provided in Figure 2A-2C.

In general, Cluster2–CMS2 and Cluster5-CMS3 displayed significantly higher epithelial cell content than Cluster3–CMS1 and Cluster4–CMS4 (Figure 2A). In contrast, tumors from Cluster3–CMS1 and Cluster4–CMS4 displayed significantly higher enrichment of tumor infiltrating cells when compared to other CRC clusters. Specifically, Cluster3–CMS1 displayed the highest leukocyte concentration (Figure 2B), whereas, Cluster4–CMS4 displayed highest level of Epcam(−) / CD45 (−) cells [endothelial cells and CAFs] (Figure 2C).

**Figure 3:**
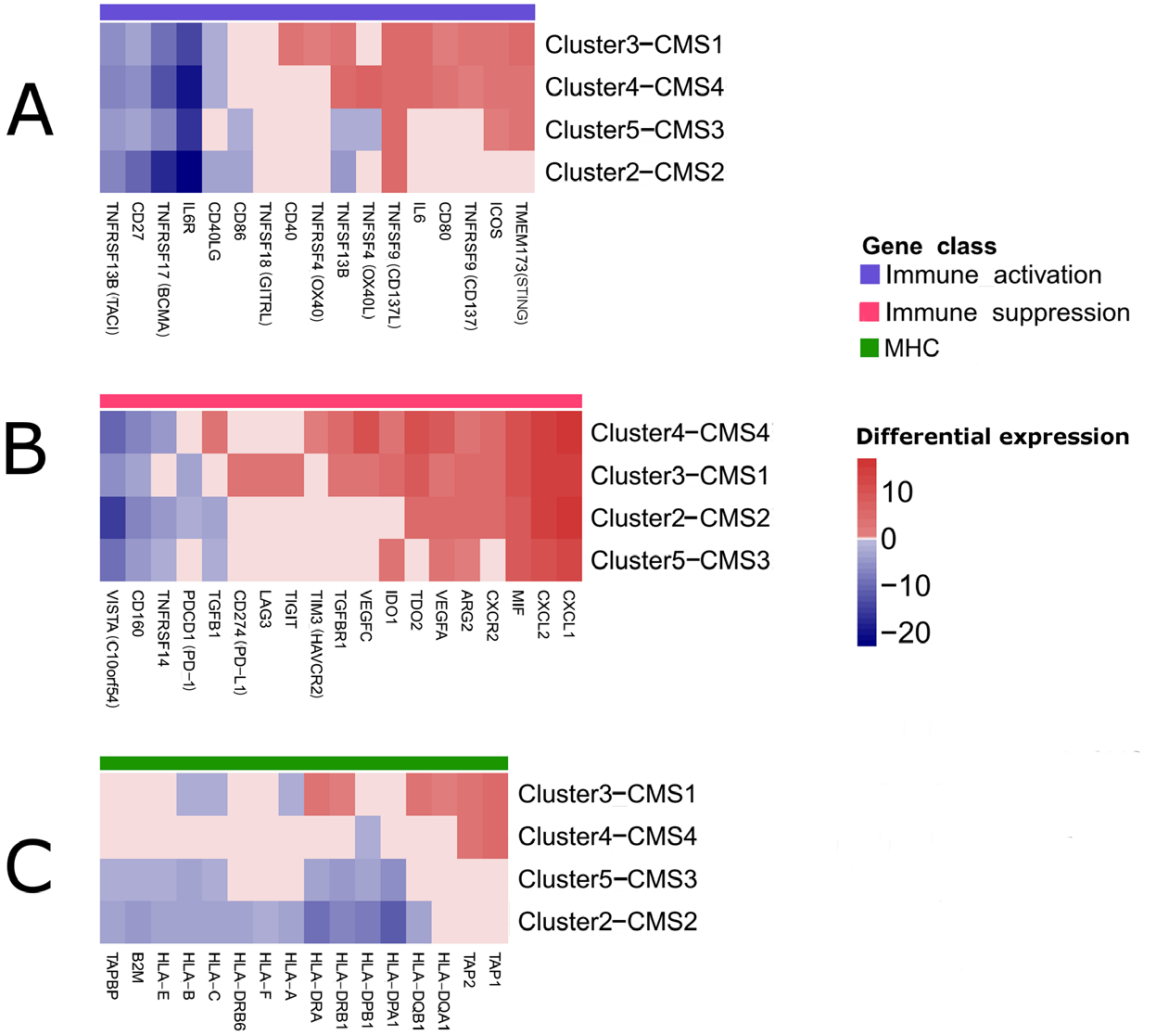
Clustered heatmaps showing selected immune modulators that were significantly deregulated in the tumor epithelial cells as revealed by csSAM [68] Both, rows and columns of the heatmaps, were clustered. For clarity, insignificant expression differences (FDR>0.05) were replaced by ‘0′ **(A)** Genes involved in immune system stimulation. **(B)** Genes involved in immune system suppression. **(C)** Major histocompatibility (MHC) genes.

In order to explore the possible effect of tumor cells on the regulation of immune influx and activity we focused on differential expression of genes in epithelial fraction of tumors. We selected 54 genes that previously have been reported to be involved in modulation of antitumor immune response (Figure 3A-3C) [5, 12].

Firstly, we addressed gene expression changes that occur in all/majority of clusters. As shown in Figure 3A *IL6R* (interleukin-6 receptor), *TNFRSF17* (*BCMA*, B cell maturation antigen), *TNFRSF13B* (*TACI*, Cyclophilin ligand interactor) and *CD27* genes, that belong to immune stimulatory class showed concordant downregulation across all CRC clusters. In at least 3 out of 4 CRC clusters we also revealed striking upregulation of genes involved in immune system activation, including *TNFSF9* (*CD137L*), *TMEM173* (*STING*) and *ICOS*. A significant fraction (40%) of genes involved in suppression in tumor microenvironment including *CXCL1*, *CXCL2*, *CXCR2*, *MIF* (macrophage migration inhibitory factor), *IDO1* (Indoleamine 2,3-dioxygenase 1), *TDO2* (TRP-2,3-dioxygenase 2), *ARG2* (arginase 2), and *VEGFA* (vascular endothelial growth factor A) displayed upregulation in at least 3 out of 4 CRC clusters (Figure 3B). On the other hand, some of them were downregulated in majority of CRC clusters (*VISTA*, *CD160* and *TNFRSF14*).

Secondly, we explored the cluster-specific gene expression changes. In general, there were significant similarities between Cluster3–CMS1 and Cluster4–CMS4 and between Cluster2-CMS2 and Cluster5–CMS3 (Figure 3A-3C). As shown in Figure 3C family of MHC genes involved in antigen processing (*TAP*) and presentation (HLA) were predominantly downregulated in Cluster2–CMS2 and Cluster5–CMS3. In cluster Cluster3–CMS1 the downregulation of HLA class I genes (*HLA-A*, *HLA-B* and *HLA-C*) was also noticeable. However, Cluster3–CMS1 was characterized also by increased expression of certain genes belonging to HLA class II family (*HLA-DQA1*, *HLA-DQB1*, *HLA-DRA*, *HLA-DRB1*) and two antigen processing genes (*TAP1* and *TAP2*), which were also noticeable in Cluster4–CMS4. Interestingly, ∼50% immune stimulatory genes were concordantly upregulated in Cluster3-CMS1 and Cluster4-CMS4 including *IL6*, *TNFRSF9*, *TNFSF9*, *CD80* and *TNFSF13B* (Figure 3A). However, Cluster3–CMS1 differed from the rest of CRC clusters by a relatively high number of upregulated members of TNFR (tumor necrosis factor receptor) family including CD40 and OX40. Finally, Cluster3–CMS1 and Cluster4–CMS4 exhibited notably higher number of genes involved in immunosupression than the remaining CRC clusters (Figure 3B). Apart from immunosuppressive genes shared by most clusters, Cluster4–CMS4 exhibited overexpression of *TIM3* (T-cell immunoglobulin mucin 3) and *TGFB1* (transforming growth factor-β1). In general, in Cluster3–CMS1 and Cluster4–CMS4 visibly more immunosuppressive genes were upregulated than in Cluster2–CMS2 and Cluster5–CMS3 [3].

## DISCUSSION

In the present study, we characterized 22 immune cell populations infiltrating colorectal tumors and defined cancer epithelial cell-specific differential gene expression. Subsequently, we placed the immune-tumor interface into a CRC consensus molecular subtype context. This led to the identification of common and cluster-specific properties of immune landscape as well as immune escape mechanisms.

In comparison to normal colon tissue the majority of clusters exhibited significant enrichment in innate as well as in adaptive immune cells. We also observed a common decrease of plasma cells, macrophages M2 and CTLs. As numerously reported, an increased concentration of activated Th, CTLs and plasma cells in tumor microenvironment is predictor of better prognosis for patients [13]. In contrast, high density of cells that promote immunosuppresion (mast cells, neutrophils and Tregs) are associated with tumor progression and reduced patient survival [14]. Taking together, our results illustrate the dichotomy (tumor promoting and anti-tumor) of immune cell populations that infiltrate CRC. In line with previous studies, we expected to observe the decrease of macrophage M1/M2 ratio in CRC [15]. However, in our study we found a contradictory association: M1 were enriched in CRC whereas M2 were depleted when compared to normal colon tissue (Supplementary Figure 5) [14, 16]. In the light of latest studies, the role of differentially polarized TAMs in CRC progression is not completely explained [17]. For example, Koelzer *et al*. provided evidence that increased M2 infiltration (but not M1) was associated with favorable clinicopathological characteristics of CRC. On the other hand Edin *et al*. reported that M1 macrophages were significantly associated with an improved prognosis [18, 19]. These conflicting results suggest that tumor associated macrophage (TAM) polarization stage in CRC is variable and correlates with the tumor microenvironment context, direct interaction of macrophages with tumor cells as well as the stage of disease.

Next, we addressed the inter-cluster differences in the immune cell influx. As expected, MSI-associated Cluster3–CMS1 displayed an increased infiltration of leukocytes related to adaptive and innate immunity [5, 20]. As has been described recently, MSI results in a higher load of neoantigens which in turn promotes increased leukocyte infiltration and immune response activation [5, 6]. Except for pro-tumoral activated mast cells and neutrophils other immune cells enriched in Cluster3–CMS1 were reported to play an anti-tumor role [10, 11]. In contrast to Cluster3–CMS1, Cluster4–CMS4 was highly infiltrated by cells showing pro-tumor activity accompanied by increased proportions of CAFs and endothelial cells. [21]. The immune infiltrate composition of Cluster4–CMS4 is likely the effect of chronic inflammation processes ongoing in this type of CRC and correlates with increased angiogenesis and EMT processes [3]. The Cluster2-CMS2 and Cluster5–CMS3 were characterized by significantly lower influx of leukocytes, CAFs and endothelial cells and reduced immune activation. In Cluster2-CMS2 we observed decreased levels of leukocytes in their active states with exception of anti-tumoral memory B cells and active T CD4 memory cells. Cluster5–CMS3 displayed low levels of immune activation except of elevated levels of active dendritic cells. These results confirm previous observations concerning Cluster2-CMS2 and Cluster5–CMS3 as a poorly immunogenic [3].

Subsequently, we focused on epithelial-specific differential expression of 54 immune related genes. We assumed that the expression or secretion of some of these proteins by CRC epithelial cells in soluble forms or by tumor-derived exosomes may create a favorable environment to support tumorigenesis by protecting tumor cells from immune anti-tumor actions [22].

In all clusters we observed a significant downregulation of several immune stimulatory genes. *TNFRSF13B* (*TACI*) and *TNFRSF17* (*BCMA*) act as B-cell stimulation factors [23, 24]. Both genes are expressed by colon epithelial cells and play a role in maturation of naive B cells into mature IgA-secreting plasma cells which is one of the crucial elements in the maintenance of intestinal immune homeostasis [25]. Given the striking decrease of plasma cells content in the tumor tissue that we observed in this study we hypothesize that this could be the result of depletion of B cells maturation signals maintained by *TACI* and *BCMA*. Significant downregulation of *IL6R* with retention/upregulation of *IL-6* expression observed in our study suggests that so called IL-6 trans-signaling plays an important role in CRC [26]. A possible meaning of IL-6 trans-signaling in CRC has been recently provided by Tseng-Rogenski *et al*. who have shown that IL-6 regulates localization of hMSH3 protein which in turn contributes to carcinogenesis by formation of microsatellite alterations at selected tetranucleotide repeats (EMAST) in 60% of CRCs [27]. This phenomenon supports our finding of prevalent retention/upregulation of IL-6 by CRC epithelial tumor cells [28]. In most clusters, we also revealed upregulation of genes involved in immune system activation. Overexpression of immunostimulatory molecules by tumor epithelial cells might seem conflicting at first glance, contrasting with numerous reports showing that in general immune stimulation generates anti-tumor T cell immune responses [29]. However, as described above for IL-6, a number of pro-tumoral roles of immunostimulators has recently emerged. Recent study by Eun *et al*. provided evidence that ligation of TNFSF9 on T cells limits their differentiation or expansion and indirectly supports generation of Tregs [30]. Huang *et al*. as well as Lemos *et al*., provided evidence that DNA sensing via STING is capable of mediating immunosupression by activation of IDO1 [31, 32]. Consistent with this notion, we observed upregulation of *STING* and *IDO1* in the same CRC clusters. Finally, recent data obtained on melanoma and breast cancer indicated that there is an association between the expression of ICOS and the production of immunosuppresive interleukin IL-10 in Tregs that results in suppression of T cells and dendritic cells [33]. Collectively, this data indicates that so called immunostimulators may play dual, context-dependent roles that also include immunosuppression. Among the genes known to be involved in immune system suppression we found 8 (out of 19 selected genes) that have been upregulated in at least 3 CRC clusters. Therefore, the secretion of immunosuppressive factors can be regarded as a common ‘strategy’ of immune escape in CRC. *CXCL1*, *CXCL2* and *CXCR2* are potent chemoattractants of immunosuppressive cells including granulocytic myeloid-derived suppressor cells (MDSCs) and neutrophils [14]. Recent studies provided evidence that *MIF* is necessary for the immunosuppressive function of TAM and MDSCs in breast cancer and melanoma [34]. *IDO1* and *TDO2* are involved in catabolism of tryptophan (trp) which depletion in tumor microenvironment results in inhibition of T cell responses. *IDO1* is currently well recognized as a potent target of anti-tumor therapies [35]. Similarly, upregulation of *ARG2* by depleting the extracellular content of arginine, induces inhibition of T cell proliferation [36]. Recently, it has been shown that *VEGFA* also exhibits immunosuppressive properties in addition to its pro-angiogenic role. *VEGFA* can induce the accumulation of tumor-associated macrophages, MDSCs, Tregs, and inhibit the migration of T lymphocytes to the tumor [37].

Next, we explored the cluster-specific gene expression changes. In general, there were significant similarities between Cluster3–CMS1 and Cluster4–CMS4 and between Cluster2-CMS2 and Cluster5–CMS3. Cluster2-CMS2 and Cluster5–CMS3 displayed widespread downregulation of MHC class genes. Downregulation of three MHC class I genes was also detected in Cluster3–CMS1. These findings are in line with reports that clearly show that by defective antigen presentation, CRC tumor cells evade immune surveillance. However, for the first time, we have demonstrated that: *a)* this phenomenon is not exhibited by all microsatellite stable CRC clusters; *b)* to some extent defective antigen presentation might be also present in MSI associated Cluster3–CMS1 [29]. Surprisingly, we noticed upregulation of antigen processing genes in Cluster3–CMS1 and Cluster4–CMS4. This was also shown by Angelova *et al*. for MSI CRCs [5]. Such a phenomenon could be explained by the evolution of Cluster3–CMS1 and Cluster4–CMS4 cancer clones in the microenvironment with high leukocyte infiltration and increased anti-tumor immune activity. As proposed by Dunn and co-workers this may lead to the elimination of highly antigenic cells and immune-based selection of tumor cells that exhibit on their surface weak/unrecognizable antigenic peptide repertoire (so called ‘immunoediting’) [38]. In such cases, in which immune surveillance is ineffective, the unchanged or even upregulated expression of MHC genes would be permitted [39]. In Cluster3–CMS1 and Cluster4–CMS4 we revealed upregulation of some genes involved in immune system activation (for example *CD80)*. CD80 (B7-1) is a costimulator mediating T-cell activation thorough CD28 receptor on T cells. However a potent immune suppressor CTLA-4 (Cytotoxic T Lymphocyte-associated Antigen-4) has much higher affinity for CD80 than CD28 [40]. CTLA-4 interaction with CD80 suppresses T cell activation and induces immunosuppressive Tregs. Given that we observed concordant upregulation of CD80 in Cluster3–CMS1 and Cluster4–CMS4 it is likely that epithelial CD80 expression enhances immunosuppression rather than immune activation in this particular context. In Cluster3–CMS1 we found relatively high number of upregulated members of TNFR (tumor necrosis factor receptor) family including *CD40* and *OX40*. All three molecules have been reported to exert T effector activity and diminish inhibitory effects of Tregs, thus they maintain antitumor immune activities [41]. A possible explanation for this contradiction may involve similar phenomenon as has been described above for IL-6, namely, expression of given TNF receptor may confer a growth advantage that is pathway-independent of the immune system. Indeed, Baxendale *et al*. have described a favorable contribution of *CD40* expression to cell transformation and neoplastic growth thorough NF-κB signalling pathway [42]. In this regard, increased secretion of CD40 from epithelial cancer cells may regulate their own properties within the tumor mass in an autocrine or paracrine fashion.

Finally, Cluster3–CMS1 and Cluster4–CMS4 exhibited notably higher number of upregulated immune suppressive genes than the remaining CRC clusters. For example, Cluster4–CMS4 exhibited specific overexpression of *TIM3* and *TGFB1*. *TIM3* is an important regulator of CTL exhaustion and apoptosis, whereas *TGFB1* is a potent immunosuppressor that downregulates the host immune response *via* multiple mechanisms including promotion of M2 macrophages and Tregs and suppression of NK cells and T CD8 [43, 44]. Upregulation of *PD-L1*, *Lag3* and *TIGIT* was specifically observed in Cluster3–CMS1. All three proteins are well characterized negative checkpoint regulators and can function synergistically to enable tumor cells to inhibit the anti-tumoral immunological activities *via* induction of T cell exhaustion and/or induction of Tregs [13, 45, 46].

In summary, our results support the existence of both commonalities and cluster-specific differences of immunological landscape and profile of immune modifiers expressed by tumor epithelial cells. We believe that commonalities between CRC clusters will have important implications for immunotherapy by allowing the development and/or improvement of therapeutic strategies for majority of CRC patients. Common anti-immune mechanisms uncovered in this study include: 1) downregulation of B-cell stimulation factors (*TACI*, *BCMA*, *CD27*) that likely results in significant depletion of plasma cells, 2) upregulation of chemoattractants of potent immunosuppressive cells (*MIF*, *CXCL1*, *CXCL2* and *CXCR2)*, 3) upregulation of enzymes (*IDO1*, *TDO2*, *ARG2*) that drain microenvironment from arginine and tryptophan which profoundly inhibit T cell proliferation, 4) defective antigen processing and presentation, which make the proper recognition of tumor cells impossible. Apart from the immune escape mechanisms characteristic for all identified clusters, we identified two additional mechanisms occurring only in Cluster3–CMS1 and Cluster4–CMS4. In Cluster3-CMS1 we observed specific upregulation of three negative checkpoint molecules (*PD-L1*, *Lag-3* and *TIGIT*) involved in regulation of T cell-mediated immunity. On the other hand, the immune escape mechanism dependent on *TGF-β1* seems to be the most important in Cluster4–CMS4. Obtained results show that effective CRC immunotherapy will require combinatorial approaches to break suppression of anti-cancer immunity. However, only knowledge on tumor transcriptional subtype will enable planning a precise and efficient anti-tumor therapy. In the light of the above, the transcriptional subtyping should be considered as a minimal requirement for developing optimal therapeutic strategies for CRC in the near future.

## MATERIALS AND METHODS

### Data collection, preprocessing and identification of transcriptional subtypes in CRC

Clinical data and Affymetrix U133 Plus 2.0 microarray expression raw files (.CEL) were collated from 15 studies which included 1597 tumor and 125 normal adjacent colon samples (Gene Expression Omnibus accession numbers: GSE69657, GSE8671, GSE9254, GSE13067, GSE13294, GSE14333, GSE17536, GSE17537, GSE18105, GSE19860, GSE28702, GSE33113, GSE35896, GSE37364 and GSE39582), see Supplementary Table 2 [47–58]. All arrays were assessed for RNA quality using the “AffyRNADegradation” package [7]. Samples with d^k^ < 0.45 [equivalent of RNA integrity measure RIN > 7] were removed from further analysis as proposed by Fasold et al. [59].

Raw data was normalized by RMA or MAS5 using the “affy” package, mapped to the NCBI Entrez gene identifiers using a custom chip definition file (Brainarray, Version 20) [60]. Outlier samples were detected and removed by principal component analysis (PCA) using RMA normalized data. Subsequently, samples that formed small batches (n ≤ 5) were removed from further analysis. We than used the ComBat algorithm implemented in the “swamp” package to correct the data for batch effects [61]. Remaining CRC samples (n = 1492) were then clustered by use of mapping of multiple clustering algorithms (COMMUNAL) approach [8]. Forty-one CRC samples were eliminated by COMMUNAL as not robustly clustered. The optimal number of K = 5 clusters was deduced based on integrative analysis of 5 clustering algorithms and 11 cluster validity metrics across the increasing variable subsets (from 1000 to 10000).

We used “ISOpureR” package to estimate normal tissue content in each sample (in each CRC cluster separately) [9, 62]. Consequently, we eliminated samples that matched low-purity Cluster 1 (see results section). In summary, after data quality control that included outlier detection, RNA-degradation, small batch removal, tumor purity estimates and lack of COMMUNAL cluster assignments, 372 tumors and 42 normal samples were removed prior to the following data analysis (see Supplementary Table 2).

### Development of microsatellite instability classifier

Given that MSI constitutes one of the most important correlates in CRC, we developed a combination of AdaBoost and random forests binary classifier using Weka 3.8 software to predict microsatellite status in 554 samples lacking this information [63]. To develop MSI classifier we used MAS5 normalized data. We included 143 MSI and 528 MSS cases and expression of top 80 genes selected by information gain attribute evaluation. We used random forests as a weak learner of AdaBoost [64]. Given the MSI/MSS class imbalance, classifier was developed in a cost sensitive mode [65]. We investigated the performance of classifier by shuffling samples and creating 20 random, independent, non-overlapping training (70% of samples) and test sets (30% of samples).

### Computation of immune cell relative proportions

MAS5 normalized data was analyzed by the CIBERSORT algorithm with 1000 iterations and the LM22 gene signature to predict relative proportions of 22 human hematopoietic cell phenotypes including naive B cells, memory B cells, plasma cells, CD8^+^ T lymphocytes (CTLs), naive CD4^+^ T lymphocytes (Th), memory resting Th, memory activated Th, follicular helper T cells (Tfh), regulatory T lymphocytes (Tregs), gamma delta T cells (γdT), resting NK cells, activated NK cells, monocytes, macrophages M0, macrophages M1, macrophages M2, resting dendritic cells (resting DCs), resting dendritic cells (activated DCs), resting mast cells, activated mast cells, eosinophils and neutrophils (see Supplementary Table 1 for details) [66].

### Characterization of tumor cellular composition and cell-specific differential gene expression

To estimate cellular composition of CRC tumors we used published expression dataset for cell populations purified from human CRCs (Gene Expression Omnibus (GEO), GSE39395) [67]. Initially, we created an expression signature using expression profiles of Epcam(+)/CD45(−) [epithelial cells], Epcam(−)/CD45(+) [leukocytes] and Epcam(−)/CD45(−) [endothelial cells and cancer-associated fibroblasts (CAFs)]. Then, we applied CIBERSORT algorithm with 1000 iterations to calculate relative proportions of cell fractions in 1225 CRCs and 83 normal colon samples [66]. Finally, relative cell proportions predicted by the CIBERSORT and gene expression profiles were used as an input to csSAM to compute cell specific differential gene expression of epithelial cells in each cluster with stringent significance threshold (FDR≤0.05) [68]. Immune-related genes (selected based on the current literature) that were significantly deregulated in tumor epithelial cells were subsequently used in the analysis of immune escape in CRC clusters [12].

### Statistical analysis

Estimated cell proportions were assessed for distribution (non-normal, normal) using the Shapiro-Wilks test (p - value ≤ 0.05) and then for comparing cell proportions between CRC clusters and normal colon tissue using nonparametric Kruskal–Wallis test or t-test [69]. We used Holm correction to adjust for multiple comparisons (adjusted p - value ≤ 0.05. Resulting odds ratios were log transformed and visualized by clustered heatmap in “NMF” package [70]. BoxPlotR tool was used to generate violin plots [71].

PK research was supported by a internal funding from the Fundation of Wroclaw Medical University.

## SUPPLEMENTARY MATERIALS FIGURES AND TABLES




