# Supplementary material for: Immunological landscape of consensus clusters in colorectal cancer
Source: Oncotarget. 2017 Oct 27;8(62):105299–311. doi: 10.18632/oncotarget.22169 (PMC5739639; doi:10.18632/oncotarget.22169)
Supplement: Supplementary file 1 [file oncotarget-08-105299-s001.pdf]

Immunological landscape of consensus clusters in colorectal cancer

SUPPLEMENTARY MATERIALS

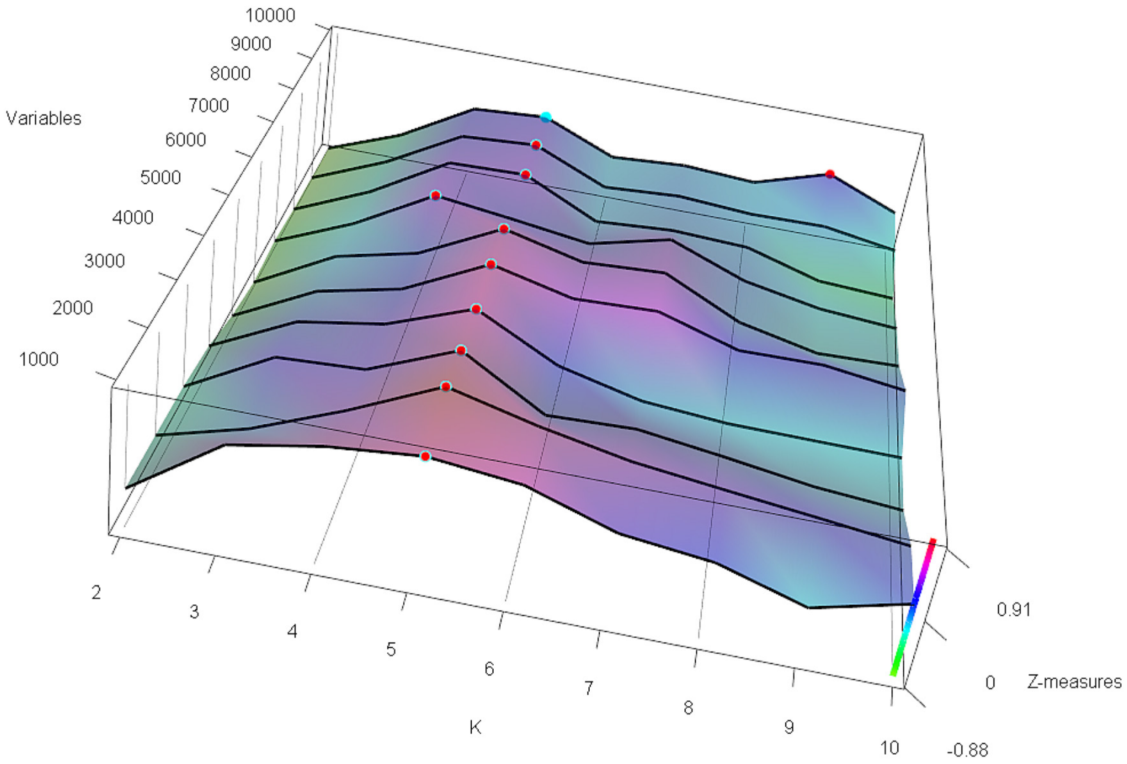

Supplementary Figure 1: 3D topographic map depicting estimation of optimal number of clusters (K) based on COMMUNAL clustering of 1492 samples. Integrative clustering has been performed by use of 5 clustering algorithms (‘hierarchical’,‘kmeans’, ‘pam’, ‘som’ and ‘agnes’) and 11 cluster validity metrics (‘Connectivity’,‘average.between’, ‘g2’, ‘avg.silwidth’, ‘average.within’, ‘dunn’, ‘widestgap’, ‘dunn2’, ‘pearsongamma’, ‘g3’ and ‘min.separation’) across the increasing variable subsets (from 1000 to 10000). We used correlation distance with an average agglomeration method. Red dot indicate potentially optimal K for a given variable subset. Note that optimal K=5 is seen in 8 out of 10 variable subsets. Please see Sweeney *et al*., for the details.

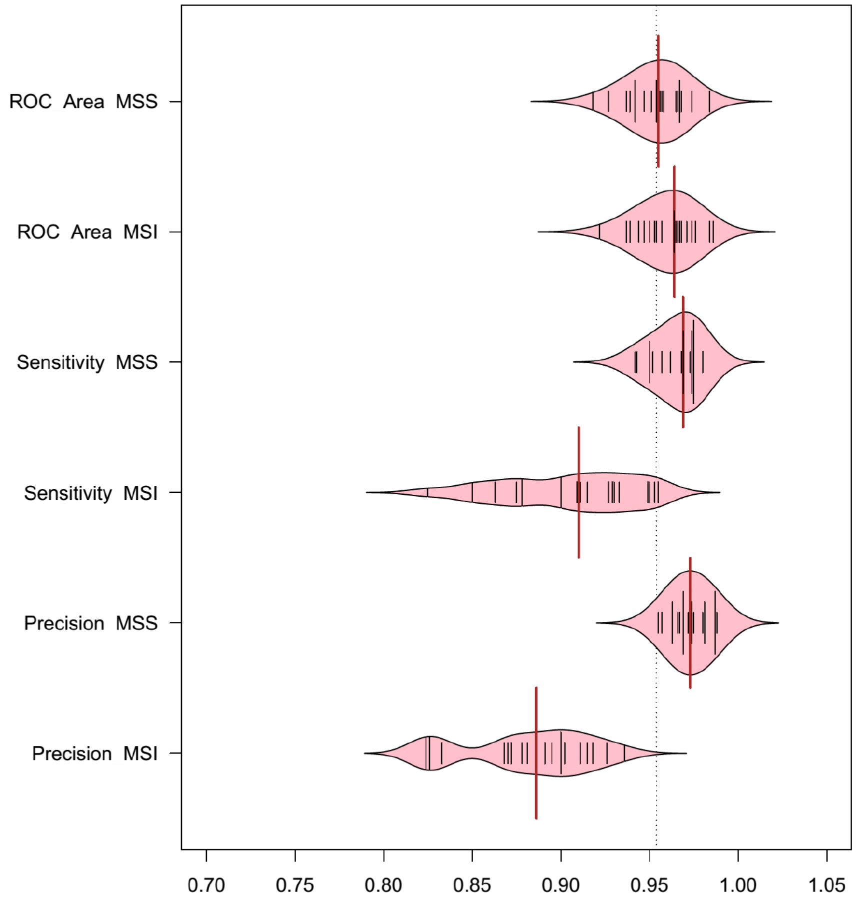

Supplementary Figure 2: Bean plots illustrating performance of MSI classifier for 20 randomly generated test sets. Sensitivity, recall and ROC curve are shown for MSI and MSS classes, respectively. Means are marked with red lines, each measurement is depicted by a black line.

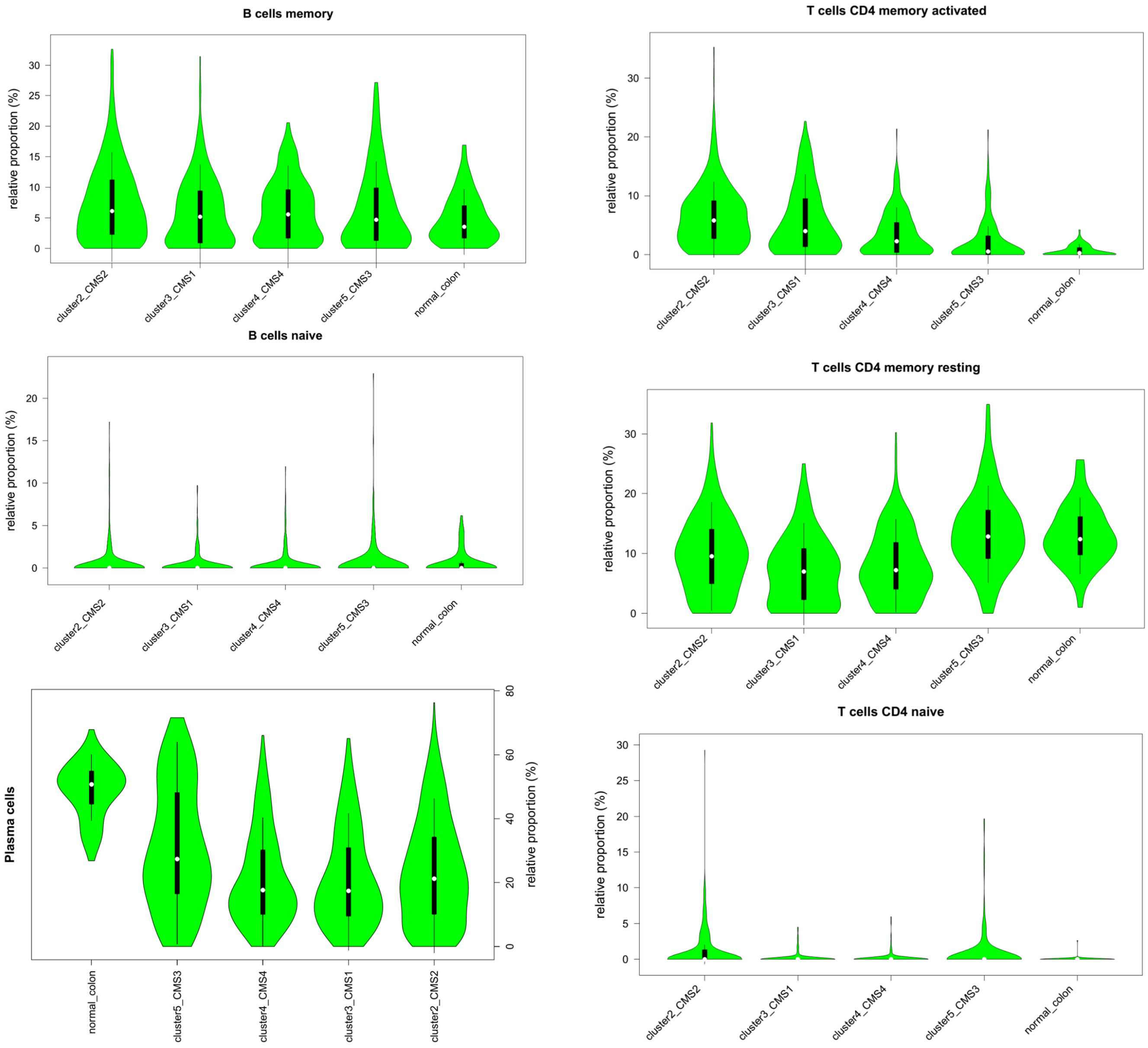
B cells memory
T cells CD4 memory activated
B cells naive
T cells CD4 memory resting
Plasma cells
T cells CD4 naive
relative proportion (%)
cluster2_CMS2
cluster3_CMS1
cluster4_CMS4
cluster5_CMS3
normal_colon

(*Continued*)

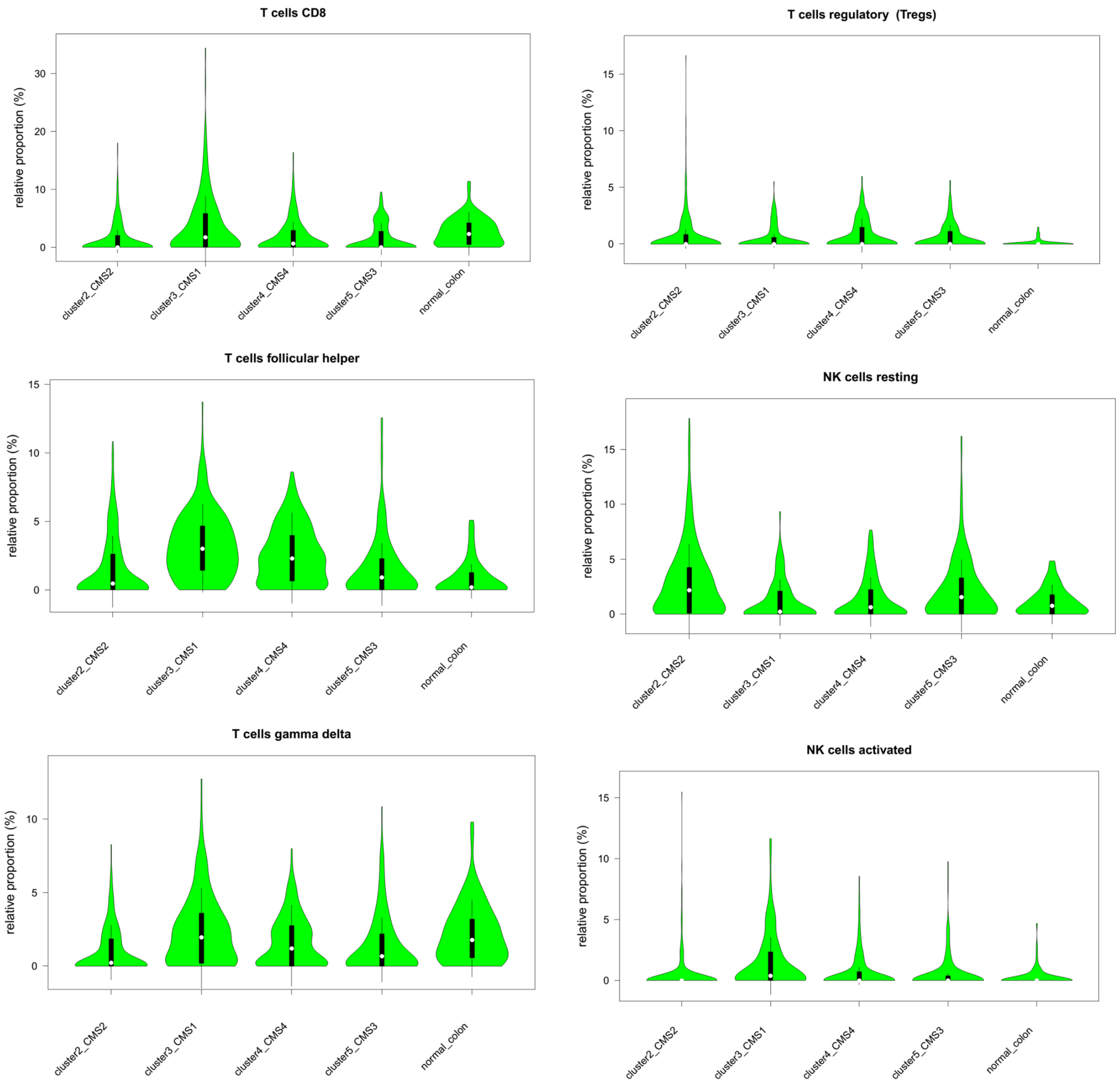
T cells CD8
T cells regulatory (Tregs)
T cells follicular helper
NK cells resting
T cells gamma delta
NK cells activated
relative proportion (%)
cluster2_CMS2
cluster3_CMS1
cluster4_CMS4
cluster5_CMS3
normal_colon

(*Continued*)

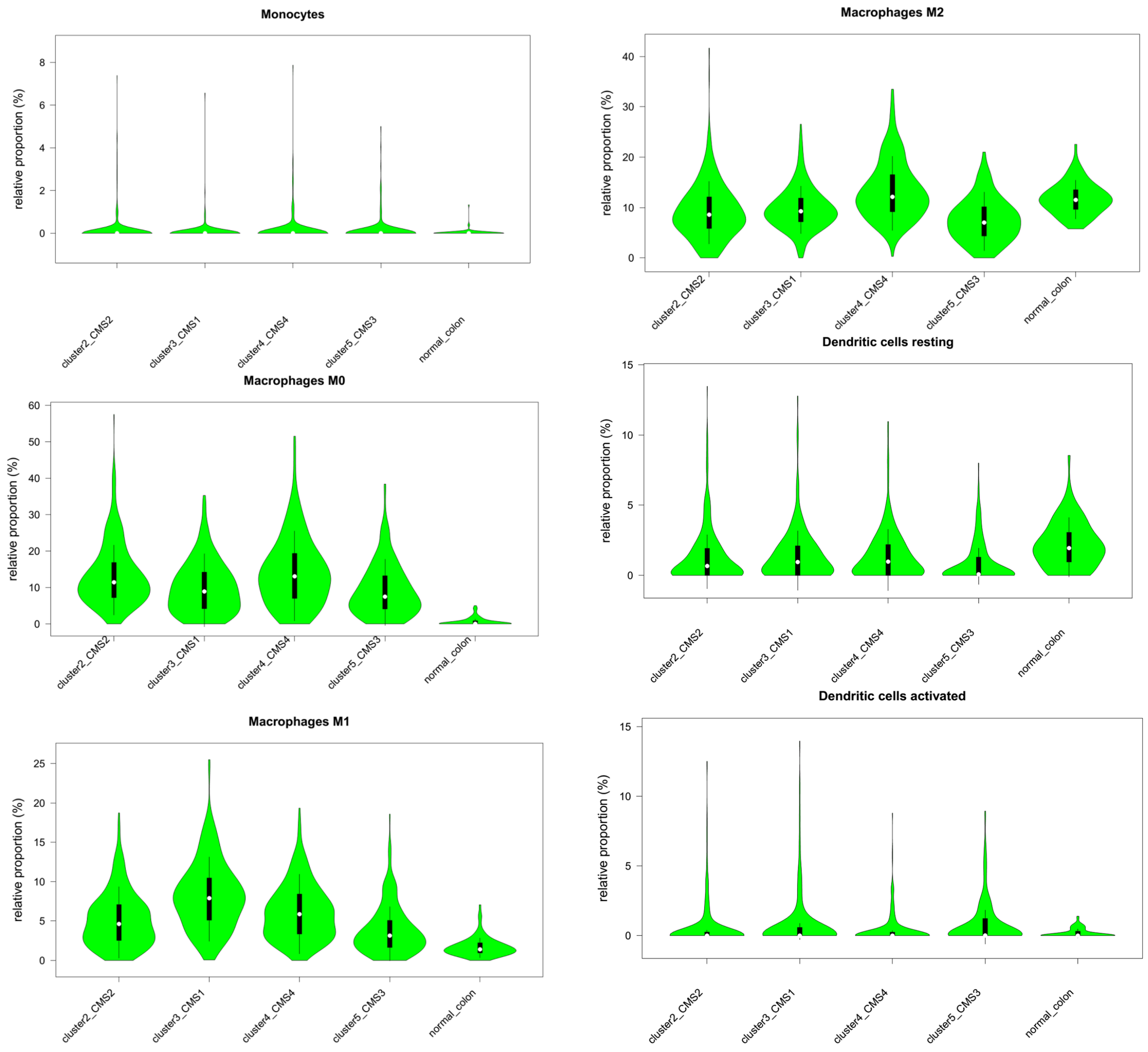
Monocytes
relative proportion (%)
0
2
4
6
8
cluster2_CMS2
cluster3_CMS1
cluster4_CMS4
cluster5_CMS3
normal_colon
Macrophages M2
relative proportion (%)
0
10
20
30
40
cluster2_CMS2
cluster3_CMS1
cluster4_CMS4
cluster5_CMS3
normal_colon
Macrophages M0
relative proportion (%)
0
10
20
30
40
50
60
cluster2_CMS2
cluster3_CMS1
cluster4_CMS4
cluster5_CMS3
normal_colon
Dendritic cells resting
relative proportion (%)
0
5
10
15
cluster2_CMS2
cluster3_CMS1
cluster4_CMS4
cluster5_CMS3
normal_colon
Macrophages M1
relative proportion (%)
0
5
10
15
20
25
cluster2_CMS2
cluster3_CMS1
cluster4_CMS4
cluster5_CMS3
normal_colon
Dendritic cells activated
relative proportion (%)
0
5
10
15
cluster2_CMS2
cluster3_CMS1
cluster4_CMS4
cluster5_CMS3
normal_colon

(*Continued*)

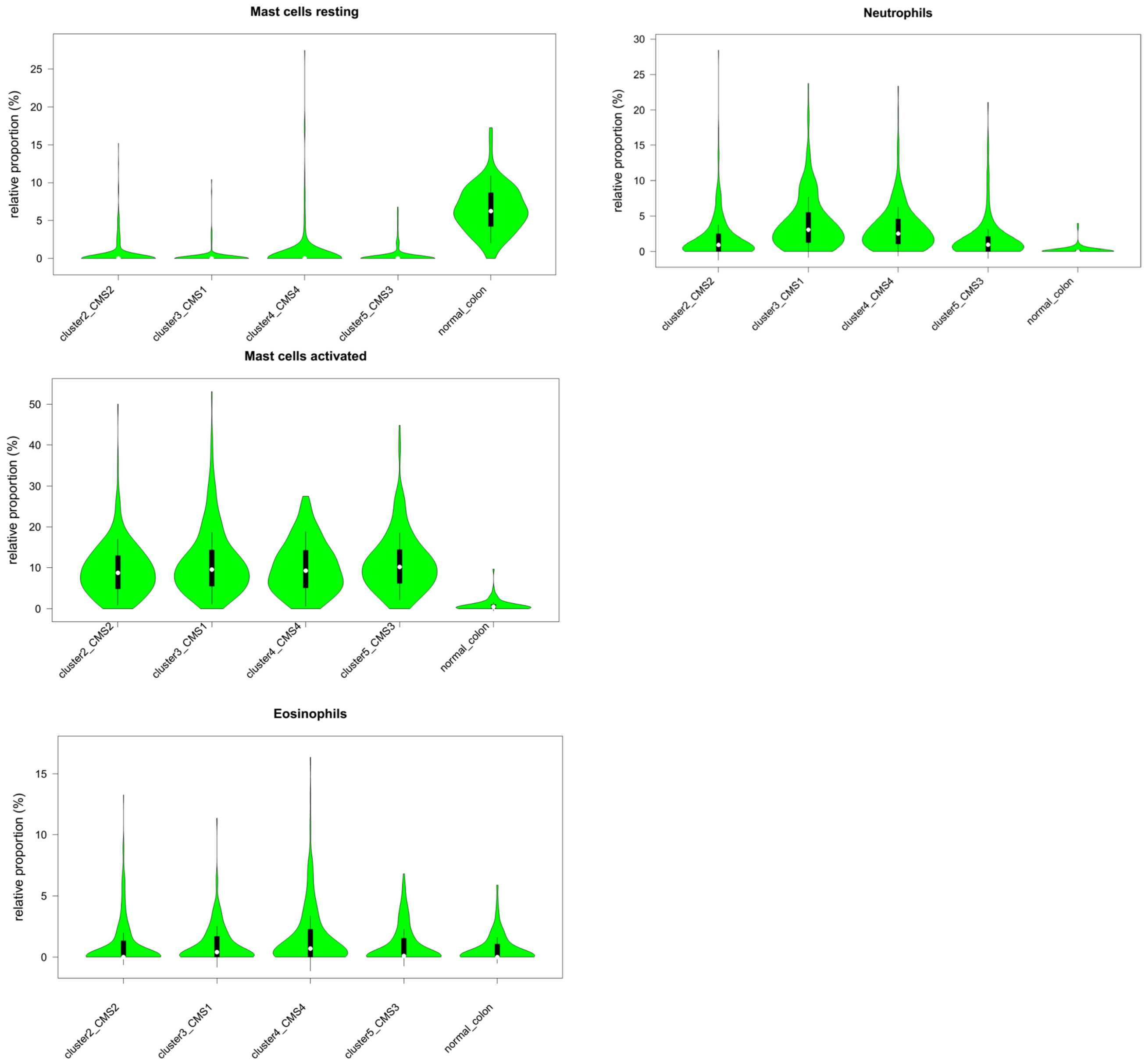

Supplementary Figure 3: Violin plots visualizing the densities of computed proportions of 22 leukocyte subpopulations in each CRC cluster and normal colon samples. Mean is marked with a white circle.

Cluster2-CMS2

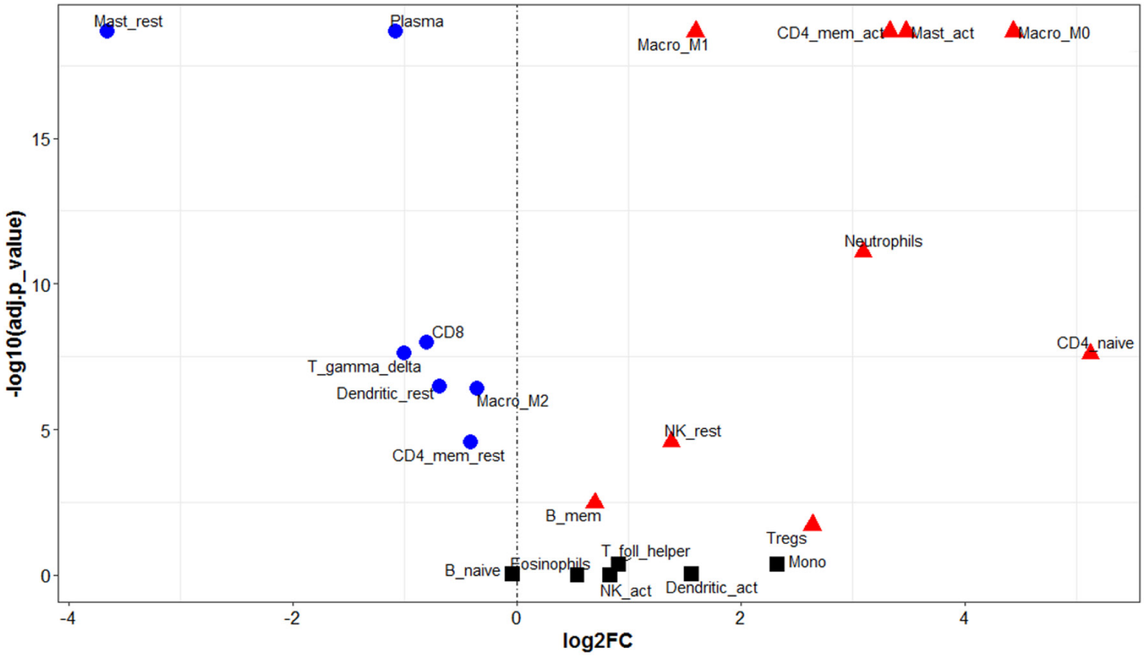

Cluster3-CMS1

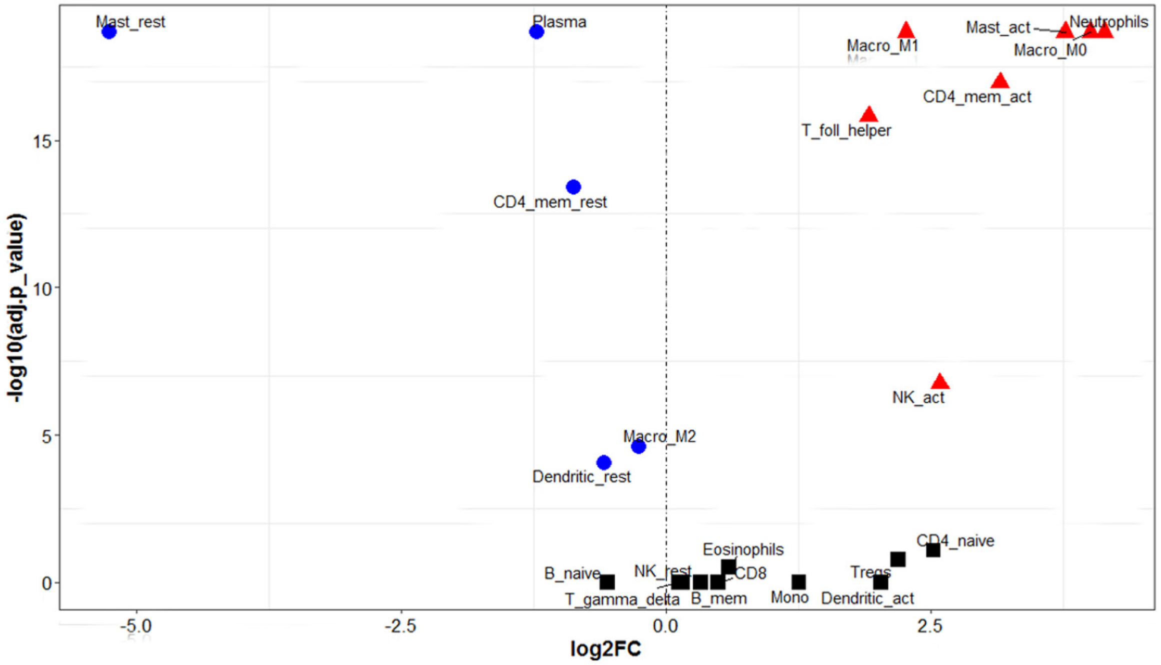

(*Continued*)

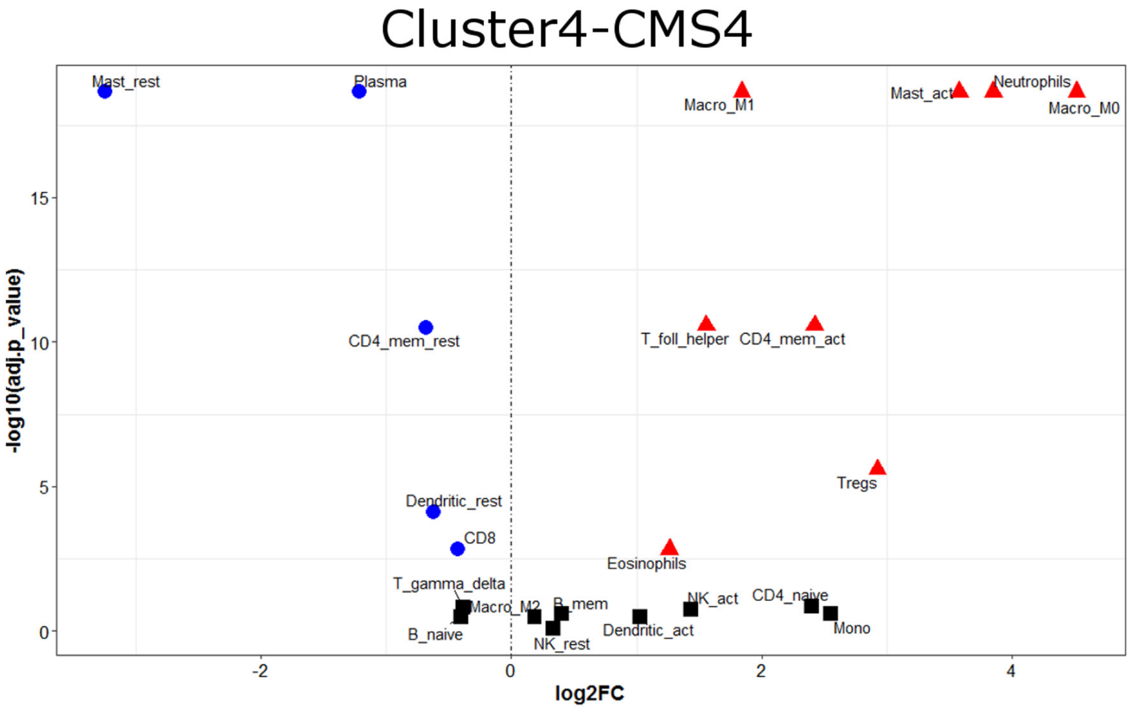

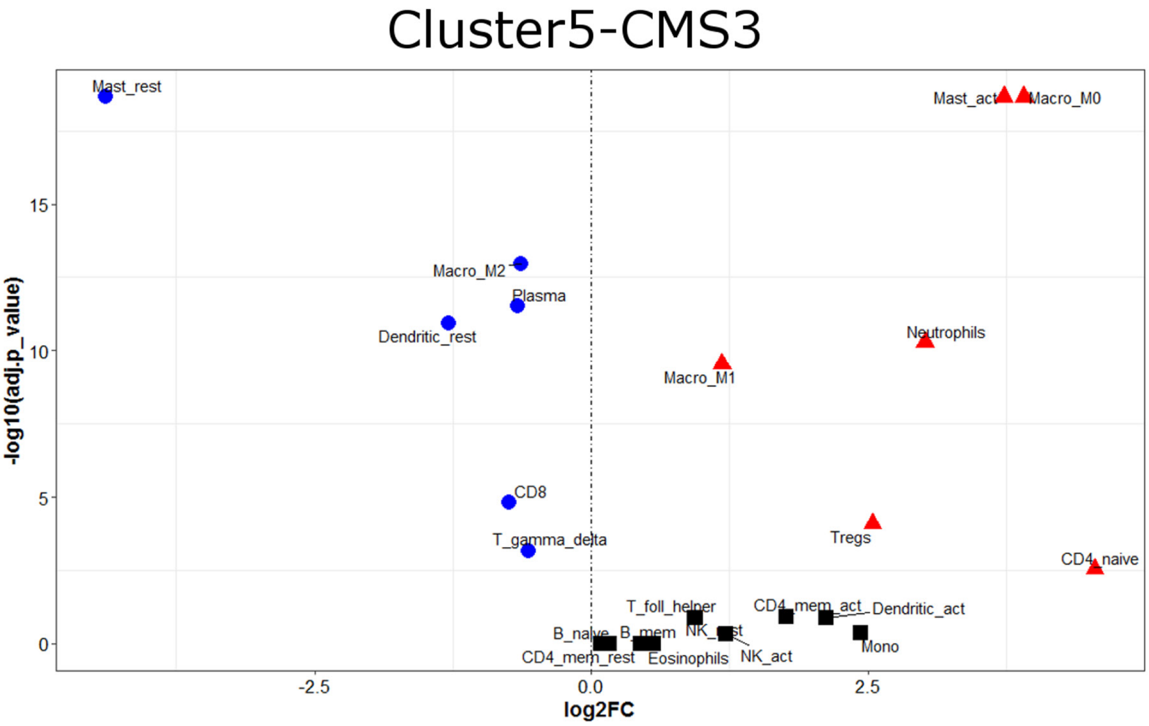

Supplementary Figure 4: Volcano plots showing average fold changes of cell proportions and statistical significance (adjusted p-value) of comparison between CRC cluster and normal colon. Blue dots represent cells that were significantly depleted in a given CRC cluster(when compared to normal samples). Black squares represents cells with insignificant changes. Red rectangles represent cells significantly enriched in a given CRC cluster (when compared to normal samples).

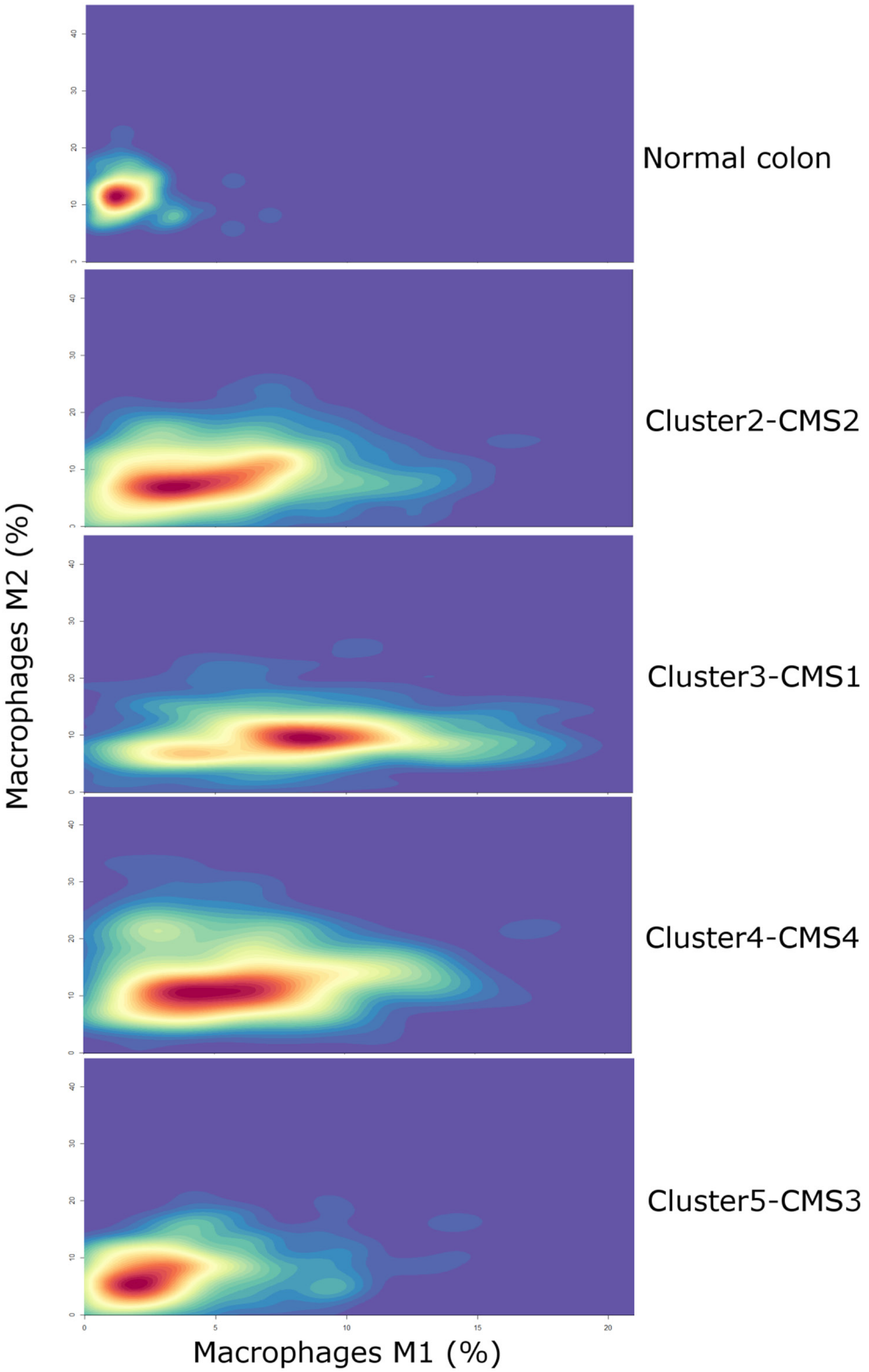

Supplementary Figure 5: 2D histograms that visualizing relation between proportions of macrophages M1 and M2 in each sample. Histograms were drawn for each CRC cluster and normal colon samples separately. Note higher content of macrophages M2 in normal tissue and a higher content of macrophages M1 in cancer tissue.

Supplementary Table 1: Detailed data on leukocytes characterized in this study

Cell Type$	Markers used$	Abbreviation used in the manuscript	Immune branch (adaptive / innate)
B cells naive	CD19+CD27- IgG/A-	naive B cells	adaptive
B cells memory	CD19+ CD27+	memory B cells	adaptive
Plasma cells	CD20+, CD138+ and CD19+	plasma cells	adaptive
T cells CD8	CD3, CD8, CD45RA	CTLs	adaptive
T cells CD4 naive	CD4+	Th	adaptive
T cells CD4 memory resting	CD45ROhigh	memory resting Th	adaptive
T cells CD4 memory activated	CD45ROhigh; CD69, CD25 for activation	memory activated Th	adaptive
T cells follicular helper	CXCR5hi, ICOShi	Tfh	adaptive
T cells regulatory	CD4+ CD25hi	Tregs	adaptive
T cells gamma delta	Not stated	gammadeltaT	innate
NK cells resting	CD56	resting NK cells	innate
NK cells activated	CD56 + CD69	activated NK cells	innate
Monocytes	N/A	monocytes	innate
Macrophages M0	None known; identified by morphology and phagocytic capability	macrophages M0	innate
Macrophages M1	None known; identified by morphology and phagocytic capability	macrophages M1	innate
Macrophages M2	None known; identified by morphology and phagocytic capability	macrophages M2	innate
Dendritic cells resting	N/A	resting DCs	innate
Dendritic cells activated	N/A	activated DCs	innate
Mast cells resting	N/A	resting mast cells	innate
Mast cells activated	N/A	activated mast cells	innate
Eosinophils	N/A	eosinophils	innate
Neutrophils	CD62L	neutrophils	innate

$ - data adopted from Newman et al., Nat Methods, 2015; 12:453-7.

Supplementary Table 2: Samples analyzed in this study

See Supplementary File 1
