# Supplementary material for: Immunological landscape of consensus clusters in colorectal cancer
Source: Oncotarget. 2017 Oct 27;8(62):105299–311. doi: 10.18632/oncotarget.22169 (PMC5739639; doi:10.18632/oncotarget.22169)
Supplement: Supplementary file 2 [file oncotarget-08-105299-s002.docx]

| **Sample_Name** | **GEO dataset** | **TNM status** | **Quality filtering inclusion/rejection status** |
| --- | --- | --- | --- |
| GSM1681353 | GSE39582 | NA | included |
| GSM1681354 | GSE39582 | NA | included |
| GSM1681355 | GSE39582 | NA | included |
| GSM1681356 | GSE39582 | NA | rejected |
| GSM1681357 | GSE39582 | NA | included |
| GSM1681358 | GSE39582 | NA | included |
| GSM1681359 | GSE39582 | NA | included |
| GSM1681360 | GSE39582 | NA | included |
| GSM1681361 | GSE39582 | NA | included |
| GSM1681362 | GSE39582 | NA | included |
| GSM1681363 | GSE39582 | NA | included |
| GSM1681364 | GSE39582 | NA | included |
| GSM1681365 | GSE39582 | NA | included |
| GSM1681366 | GSE39582 | NA | included |
| GSM1681367 | GSE39582 | NA | included |
| GSM1681368 | GSE39582 | NA | included |
| GSM1681369 | GSE39582 | NA | included |
| GSM1681370 | GSE39582 | NA | included |
| GSM1681371 | GSE39582 | NA | included |
| GSM1705389 | GSE69657 | NA | rejected |
| GSM1705390 | GSE69657 | NA | rejected |
| GSM1705391 | GSE69657 | NA | included |
| GSM1705392 | GSE69657 | NA | rejected |
| GSM1705393 | GSE69657 | NA | included |
| GSM1705394 | GSE69657 | NA | included |
| GSM1705395 | GSE69657 | NA | included |
| GSM1705396 | GSE69657 | NA | included |
| GSM1705397 | GSE69657 | NA | included |
| GSM1705398 | GSE69657 | NA | included |
| GSM1705399 | GSE69657 | NA | rejected |
| GSM1705400 | GSE69657 | NA | rejected |
| GSM1705401 | GSE69657 | NA | included |
| GSM1705402 | GSE69657 | NA | rejected |
| GSM1705403 | GSE69657 | NA | included |
| GSM1705404 | GSE69657 | NA | rejected |
| GSM215051 | GSE8671 | NA | rejected |
| GSM215052 | GSE8671 | NA | included |
| GSM215053 | GSE8671 | NA | included |
| GSM215054 | GSE8671 | NA | included |
| GSM215055 | GSE8671 | NA | included |
| GSM215056 | GSE8671 | NA | included |
| GSM215057 | GSE8671 | NA | included |
| GSM215058 | GSE8671 | NA | included |
| GSM215059 | GSE8671 | NA | included |
| GSM215060 | GSE8671 | NA | included |
| GSM215061 | GSE8671 | NA | rejected |
| GSM215062 | GSE8671 | NA | included |
| GSM215063 | GSE8671 | NA | rejected |
| GSM215064 | GSE8671 | NA | included |
| GSM215065 | GSE8671 | NA | included |
| GSM215066 | GSE8671 | NA | included |
| GSM215067 | GSE8671 | NA | included |
| GSM215068 | GSE8671 | NA | included |
| GSM215069 | GSE8671 | NA | included |
| GSM215070 | GSE8671 | NA | included |
| GSM215071 | GSE8671 | NA | included |
| GSM215072 | GSE8671 | NA | included |
| GSM215073 | GSE8671 | NA | included |
| GSM215074 | GSE8671 | NA | included |
| GSM215075 | GSE8671 | NA | included |
| GSM215076 | GSE8671 | NA | included |
| GSM215077 | GSE8671 | NA | included |
| GSM215078 | GSE8671 | NA | included |
| GSM215079 | GSE8671 | NA | included |
| GSM215080 | GSE8671 | NA | included |
| GSM215081 | GSE8671 | NA | included |
| GSM215082 | GSE9254 | NA | rejected |
| GSM234909 | GSE9254 | IIB/IIC | rejected |
| GSM234910 | GSE9254 | NA | rejected |
| GSM234911 | GSE9254 | NA | rejected |
| GSM234912 | GSE9254 | NA | rejected |
| GSM234913 | GSE9254 | NA | rejected |
| GSM234914 | GSE9254 | IV | rejected |
| GSM234915 | GSE9254 | NA | rejected |
| GSM234916 | GSE9254 | IIA | rejected |
| GSM234917 | GSE9254 | NA | rejected |
| GSM234918 | GSE9254 | NA | rejected |
| GSM234919 | GSE9254 | NA | rejected |
| GSM234920 | GSE9254 | NA | rejected |
| GSM234921 | GSE9254 | IIB/IIC | rejected |
| GSM234922 | GSE9254 | IIIB | rejected |
| GSM234923 | GSE9254 | NA | rejected |
| GSM234924 | GSE9254 | NA | rejected |
| GSM234925 | GSE9254 | IIIC | rejected |
| GSM234926 | GSE9254 | NA | rejected |
| GSM234927 | GSE9254 | NA | rejected |
| GSM327282 | GSE13067 | NA | included |
| GSM327283 | GSE13067 | NA | included |
| GSM327284 | GSE13067 | NA | included |
| GSM327285 | GSE13067 | NA | included |
| GSM327286 | GSE13067 | NA | rejected |
| GSM327287 | GSE13067 | NA | included |
| GSM327288 | GSE13067 | NA | rejected |
| GSM327289 | GSE13067 | NA | rejected |
| GSM327290 | GSE13067 | NA | included |
| GSM327291 | GSE13067 | NA | rejected |
| GSM327292 | GSE13067 | NA | included |
| GSM327293 | GSE13067 | NA | rejected |
| GSM327294 | GSE13067 | NA | included |
| GSM327295 | GSE13067 | NA | included |
| GSM327296 | GSE13067 | NA | included |
| GSM327297 | GSE13067 | NA | included |
| GSM327298 | GSE13067 | NA | rejected |
| GSM327299 | GSE13067 | NA | included |
| GSM327300 | GSE13067 | NA | rejected |
| GSM327301 | GSE13067 | NA | included |
| GSM327302 | GSE13067 | NA | rejected |
| GSM327303 | GSE13067 | NA | rejected |
| GSM327304 | GSE13067 | NA | rejected |
| GSM327305 | GSE13067 | NA | included |
| GSM327306 | GSE13067 | NA | included |
| GSM327307 | GSE13067 | NA | included |
| GSM327308 | GSE13067 | NA | included |
| GSM327309 | GSE13067 | NA | rejected |
| GSM327310 | GSE13067 | NA | included |
| GSM327311 | GSE13067 | NA | included |
| GSM327312 | GSE13067 | NA | included |
| GSM327313 | GSE13067 | NA | included |
| GSM327314 | GSE13067 | NA | rejected |
| GSM327315 | GSE13067 | NA | rejected |
| GSM327316 | GSE13067 | NA | included |
| GSM327317 | GSE13067 | NA | included |
| GSM327318 | GSE13067 | NA | included |
| GSM327319 | GSE13067 | NA | included |
| GSM327320 | GSE13067 | NA | included |
| GSM327321 | GSE13067 | NA | included |
| GSM327322 | GSE13067 | NA | included |
| GSM327323 | GSE13067 | NA | included |
| GSM327324 | GSE13067 | NA | included |
| GSM327325 | GSE13067 | NA | included |
| GSM327326 | GSE13067 | NA | included |
| GSM327327 | GSE13067 | NA | rejected |
| GSM327328 | GSE13067 | NA | rejected |
| GSM327329 | GSE13067 | NA | included |
| GSM327330 | GSE13067 | NA | rejected |
| GSM327331 | GSE13067 | NA | included |
| GSM327332 | GSE13067 | NA | rejected |
| GSM327333 | GSE13067 | NA | rejected |
| GSM327334 | GSE13067 | NA | included |
| GSM327335 | GSE13067 | NA | included |
| GSM327336 | GSE13067 | NA | rejected |
| GSM327337 | GSE13067 | NA | included |
| GSM327338 | GSE13067 | NA | included |
| GSM327339 | GSE13067 | NA | included |
| GSM327340 | GSE13067 | NA | rejected |
| GSM327341 | GSE13067 | NA | included |
| GSM327342 | GSE13067 | NA | included |
| GSM327343 | GSE13067 | NA | rejected |
| GSM327344 | GSE13067 | NA | included |
| GSM327345 | GSE13067 | NA | included |
| GSM327346 | GSE13067 | NA | included |
| GSM327347 | GSE13067 | NA | included |
| GSM327348 | GSE13067 | NA | included |
| GSM327349 | GSE13067 | NA | included |
| GSM327350 | GSE13067 | NA | included |
| GSM327351 | GSE13067 | NA | included |
| GSM327352 | GSE13067 | NA | included |
| GSM327353 | GSE13067 | NA | included |
| GSM327354 | GSE13067 | NA | included |
| GSM327355 | GSE13067 | NA | included |
| GSM335510 | GSE13294 | NA | included |
| GSM335511 | GSE13294 | NA | rejected |
| GSM335512 | GSE13294 | NA | included |
| GSM335513 | GSE13294 | NA | rejected |
| GSM335514 | GSE13294 | NA | included |
| GSM335515 | GSE13294 | NA | included |
| GSM335516 | GSE13294 | NA | included |
| GSM335517 | GSE13294 | NA | included |
| GSM335518 | GSE13294 | NA | rejected |
| GSM335519 | GSE13294 | NA | rejected |
| GSM335520 | GSE13294 | NA | rejected |
| GSM335521 | GSE13294 | NA | rejected |
| GSM335522 | GSE13294 | NA | included |
| GSM335523 | GSE13294 | NA | included |
| GSM335524 | GSE13294 | NA | included |
| GSM335525 | GSE13294 | NA | rejected |
| GSM335526 | GSE13294 | NA | rejected |
| GSM335527 | GSE13294 | NA | rejected |
| GSM335528 | GSE13294 | NA | rejected |
| GSM335529 | GSE13294 | NA | included |
| GSM335530 | GSE13294 | NA | included |
| GSM335531 | GSE13294 | NA | rejected |
| GSM335532 | GSE13294 | NA | included |
| GSM335533 | GSE13294 | NA | included |
| GSM335534 | GSE13294 | NA | included |
| GSM335535 | GSE13294 | NA | rejected |
| GSM335536 | GSE13294 | NA | rejected |
| GSM335537 | GSE13294 | NA | rejected |
| GSM335538 | GSE13294 | NA | included |
| GSM335539 | GSE13294 | NA | included |
| GSM335540 | GSE13294 | NA | included |
| GSM335541 | GSE13294 | NA | rejected |
| GSM335542 | GSE13294 | NA | rejected |
| GSM335543 | GSE13294 | NA | rejected |
| GSM335544 | GSE13294 | NA | included |
| GSM335545 | GSE13294 | NA | included |
| GSM335546 | GSE13294 | NA | rejected |
| GSM335547 | GSE13294 | NA | rejected |
| GSM335548 | GSE13294 | NA | rejected |
| GSM335549 | GSE13294 | NA | included |
| GSM335550 | GSE13294 | NA | included |
| GSM335551 | GSE13294 | NA | rejected |
| GSM335552 | GSE13294 | NA | rejected |
| GSM335553 | GSE13294 | NA | rejected |
| GSM335554 | GSE13294 | NA | included |
| GSM335555 | GSE13294 | NA | included |
| GSM335556 | GSE13294 | NA | included |
| GSM335557 | GSE13294 | NA | included |
| GSM335558 | GSE13294 | NA | included |
| GSM335559 | GSE13294 | NA | included |
| GSM335560 | GSE13294 | NA | included |
| GSM335561 | GSE13294 | NA | included |
| GSM335562 | GSE13294 | NA | included |
| GSM335563 | GSE13294 | NA | rejected |
| GSM335564 | GSE13294 | NA | rejected |
| GSM335565 | GSE13294 | NA | included |
| GSM335566 | GSE13294 | NA | included |
| GSM335567 | GSE13294 | NA | rejected |
| GSM335568 | GSE13294 | NA | included |
| GSM335569 | GSE13294 | NA | rejected |
| GSM335570 | GSE13294 | NA | rejected |
| GSM335571 | GSE13294 | NA | rejected |
| GSM335572 | GSE13294 | NA | included |
| GSM335573 | GSE13294 | NA | rejected |
| GSM335574 | GSE13294 | NA | included |
| GSM335575 | GSE13294 | NA | rejected |
| GSM335576 | GSE13294 | NA | included |
| GSM335577 | GSE13294 | NA | included |
| GSM335578 | GSE13294 | NA | rejected |
| GSM335579 | GSE13294 | NA | included |
| GSM335580 | GSE13294 | NA | included |
| GSM335581 | GSE13294 | NA | included |
| GSM335582 | GSE13294 | NA | included |
| GSM335583 | GSE13294 | NA | included |
| GSM335584 | GSE13294 | NA | included |
| GSM335585 | GSE13294 | NA | rejected |
| GSM335586 | GSE13294 | NA | included |
| GSM335587 | GSE13294 | NA | included |
| GSM335588 | GSE13294 | NA | included |
| GSM335589 | GSE13294 | NA | included |
| GSM335590 | GSE13294 | NA | rejected |
| GSM335591 | GSE13294 | NA | included |
| GSM335592 | GSE13294 | NA | included |
| GSM335593 | GSE13294 | NA | included |
| GSM335594 | GSE13294 | NA | included |
| GSM335595 | GSE13294 | NA | rejected |
| GSM335596 | GSE13294 | NA | rejected |
| GSM335597 | GSE13294 | NA | rejected |
| GSM335598 | GSE13294 | NA | rejected |
| GSM335599 | GSE13294 | NA | included |
| GSM335600 | GSE13294 | NA | rejected |
| GSM335601 | GSE13294 | NA | included |
| GSM335602 | GSE13294 | NA | rejected |
| GSM335603 | GSE13294 | NA | included |
| GSM335604 | GSE13294 | NA | rejected |
| GSM335605 | GSE13294 | NA | included |
| GSM335606 | GSE13294 | NA | included |
| GSM335607 | GSE13294 | NA | included |
| GSM335608 | GSE13294 | NA | included |
| GSM335609 | GSE13294 | NA | included |
| GSM335610 | GSE13294 | NA | included |
| GSM335611 | GSE13294 | NA | included |
| GSM335612 | GSE13294 | NA | included |
| GSM335613 | GSE13294 | NA | included |
| GSM335614 | GSE13294 | NA | included |
| GSM335615 | GSE13294 | NA | included |
| GSM335616 | GSE13294 | NA | rejected |
| GSM335617 | GSE13294 | NA | included |
| GSM335618 | GSE13294 | NA | included |
| GSM335619 | GSE13294 | NA | included |
| GSM335620 | GSE13294 | NA | rejected |
| GSM335621 | GSE13294 | NA | included |
| GSM335622 | GSE13294 | NA | included |
| GSM335623 | GSE13294 | NA | included |
| GSM335624 | GSE13294 | NA | included |
| GSM335625 | GSE13294 | NA | rejected |
| GSM335626 | GSE13294 | NA | included |
| GSM335627 | GSE13294 | NA | included |
| GSM335628 | GSE13294 | NA | included |
| GSM335629 | GSE13294 | NA | included |
| GSM335630 | GSE13294 | NA | included |
| GSM335631 | GSE13294 | NA | included |
| GSM335632 | GSE13294 | NA | included |
| GSM335633 | GSE13294 | NA | rejected |
| GSM335634 | GSE13294 | NA | rejected |
| GSM335635 | GSE13294 | NA | included |
| GSM335636 | GSE13294 | NA | included |
| GSM335637 | GSE13294 | NA | rejected |
| GSM335638 | GSE13294 | NA | rejected |
| GSM335639 | GSE13294 | NA | included |
| GSM335640 | GSE13294 | NA | included |
| GSM335641 | GSE13294 | NA | rejected |
| GSM335642 | GSE13294 | NA | rejected |
| GSM335643 | GSE13294 | NA | included |
| GSM335644 | GSE13294 | NA | included |
| GSM335645 | GSE13294 | NA | included |
| GSM335646 | GSE13294 | NA | rejected |
| GSM335647 | GSE13294 | NA | rejected |
| GSM335648 | GSE13294 | NA | included |
| GSM335649 | GSE13294 | NA | included |
| GSM335650 | GSE13294 | NA | included |
| GSM335651 | GSE13294 | NA | included |
| GSM335652 | GSE13294 | NA | included |
| GSM335653 | GSE13294 | NA | included |
| GSM335654 | GSE13294 | NA | included |
| GSM335655 | GSE13294 | NA | included |
| GSM335656 | GSE13294 | NA | rejected |
| GSM335657 | GSE13294 | NA | included |
| GSM335658 | GSE13294 | NA | included |
| GSM335659 | GSE13294 | NA | rejected |
| GSM335660 | GSE13294 | NA | included |
| GSM335661 | GSE13294 | NA | rejected |
| GSM335662 | GSE13294 | NA | included |
| GSM335663 | GSE13294 | NA | rejected |
| GSM335664 | GSE13294 | NA | included |
| GSM358341 | GSE14333 | NA | included |
| GSM358342 | GSE14333 | NA | included |
| GSM358343 | GSE14333 | NA | rejected |
| GSM358344 | GSE14333 | NA | included |
| GSM358345 | GSE14333 | NA | included |
| GSM358346 | GSE14333 | NA | included |
| GSM358347 | GSE14333 | NA | included |
| GSM358348 | GSE14333 | NA | included |
| GSM358349 | GSE14333 | NA | included |
| GSM358350 | GSE14333 | NA | included |
| GSM358351 | GSE14333 | NA | included |
| GSM358352 | GSE14333 | NA | included |
| GSM358353 | GSE14333 | NA | included |
| GSM358354 | GSE14333 | NA | included |
| GSM358355 | GSE14333 | NA | included |
| GSM358356 | GSE14333 | NA | included |
| GSM358357 | GSE14333 | NA | included |
| GSM358358 | GSE14333 | NA | included |
| GSM358359 | GSE14333 | NA | included |
| GSM358360 | GSE14333 | NA | included |
| GSM358361 | GSE14333 | NA | included |
| GSM358362 | GSE14333 | NA | included |
| GSM358363 | GSE14333 | NA | included |
| GSM358364 | GSE14333 | NA | included |
| GSM358365 | GSE14333 | NA | included |
| GSM358366 | GSE14333 | NA | included |
| GSM358367 | GSE14333 | NA | included |
| GSM358368 | GSE14333 | NA | rejected |
| GSM358369 | GSE14333 | NA | included |
| GSM358370 | GSE14333 | NA | included |
| GSM358371 | GSE14333 | NA | included |
| GSM358372 | GSE14333 | NA | included |
| GSM358373 | GSE14333 | NA | included |
| GSM358374 | GSE14333 | NA | rejected |
| GSM358375 | GSE14333 | NA | included |
| GSM358376 | GSE14333 | NA | included |
| GSM358377 | GSE14333 | NA | included |
| GSM358378 | GSE14333 | NA | included |
| GSM358379 | GSE14333 | NA | rejected |
| GSM358380 | GSE14333 | NA | included |
| GSM358381 | GSE14333 | NA | rejected |
| GSM358382 | GSE14333 | NA | included |
| GSM358383 | GSE14333 | NA | included |
| GSM358384 | GSE14333 | NA | rejected |
| GSM358385 | GSE14333 | NA | included |
| GSM358386 | GSE14333 | NA | included |
| GSM358387 | GSE14333 | NA | included |
| GSM358388 | GSE14333 | NA | included |
| GSM358389 | GSE14333 | NA | included |
| GSM358390 | GSE14333 | NA | included |
| GSM358391 | GSE14333 | NA | included |
| GSM358392 | GSE14333 | NA | included |
| GSM358393 | GSE14333 | NA | included |
| GSM358394 | GSE14333 | NA | included |
| GSM358395 | GSE14333 | NA | included |
| GSM358396 | GSE14333 | NA | included |
| GSM358397 | GSE14333 | NA | included |
| GSM358398 | GSE14333 | NA | included |
| GSM358399 | GSE14333 | NA | included |
| GSM358400 | GSE14333 | NA | included |
| GSM358401 | GSE14333 | NA | included |
| GSM358402 | GSE14333 | NA | included |
| GSM358403 | GSE14333 | NA | rejected |
| GSM358404 | GSE14333 | NA | included |
| GSM358405 | GSE14333 | NA | included |
| GSM358406 | GSE14333 | NA | rejected |
| GSM358407 | GSE14333 | NA | included |
| GSM358408 | GSE14333 | NA | included |
| GSM358409 | GSE14333 | NA | rejected |
| GSM358410 | GSE14333 | NA | included |
| GSM358411 | GSE14333 | NA | rejected |
| GSM358412 | GSE14333 | NA | included |
| GSM358413 | GSE14333 | NA | included |
| GSM358414 | GSE14333 | NA | included |
| GSM358415 | GSE14333 | NA | included |
| GSM358416 | GSE14333 | NA | included |
| GSM358417 | GSE14333 | NA | included |
| GSM358418 | GSE14333 | NA | included |
| GSM358419 | GSE14333 | NA | included |
| GSM358420 | GSE14333 | NA | included |
| GSM358421 | GSE14333 | NA | rejected |
| GSM358422 | GSE14333 | NA | included |
| GSM358423 | GSE14333 | NA | included |
| GSM358424 | GSE14333 | NA | included |
| GSM358425 | GSE14333 | NA | included |
| GSM358426 | GSE14333 | NA | rejected |
| GSM358427 | GSE14333 | NA | rejected |
| GSM358428 | GSE14333 | NA | included |
| GSM358429 | GSE14333 | NA | included |
| GSM358430 | GSE14333 | NA | included |
| GSM358431 | GSE14333 | NA | included |
| GSM358432 | GSE14333 | NA | included |
| GSM358433 | GSE14333 | NA | included |
| GSM358434 | GSE14333 | NA | included |
| GSM358435 | GSE14333 | NA | rejected |
| GSM358436 | GSE14333 | NA | rejected |
| GSM358437 | GSE14333 | NA | included |
| GSM358438 | GSE14333 | NA | included |
| GSM358439 | GSE14333 | NA | included |
| GSM358440 | GSE14333 | NA | rejected |
| GSM358441 | GSE14333 | NA | included |
| GSM358442 | GSE14333 | NA | included |
| GSM358443 | GSE14333 | NA | included |
| GSM358444 | GSE14333 | NA | rejected |
| GSM358445 | GSE14333 | NA | included |
| GSM358446 | GSE14333 | NA | included |
| GSM358447 | GSE14333 | NA | included |
| GSM358448 | GSE14333 | NA | included |
| GSM358449 | GSE14333 | NA | included |
| GSM358450 | GSE14333 | NA | rejected |
| GSM358451 | GSE14333 | NA | included |
| GSM358452 | GSE14333 | NA | rejected |
| GSM358453 | GSE14333 | NA | rejected |
| GSM358454 | GSE14333 | NA | included |
| GSM358455 | GSE14333 | NA | included |
| GSM358456 | GSE14333 | NA | included |
| GSM358457 | GSE14333 | NA | included |
| GSM358458 | GSE14333 | NA | included |
| GSM358459 | GSE14333 | NA | included |
| GSM358460 | GSE14333 | NA | included |
| GSM358461 | GSE14333 | NA | included |
| GSM358462 | GSE14333 | NA | rejected |
| GSM358463 | GSE14333 | NA | included |
| GSM358464 | GSE14333 | NA | included |
| GSM358465 | GSE14333 | NA | rejected |
| GSM358466 | GSE14333 | NA | included |
| GSM358467 | GSE14333 | NA | included |
| GSM358468 | GSE14333 | NA | rejected |
| GSM358469 | GSE14333 | NA | included |
| GSM358470 | GSE14333 | NA | included |
| GSM358471 | GSE14333 | NA | included |
| GSM358472 | GSE14333 | NA | included |
| GSM358473 | GSE14333 | NA | included |
| GSM358474 | GSE14333 | NA | included |
| GSM358475 | GSE14333 | NA | included |
| GSM358476 | GSE14333 | NA | rejected |
| GSM358477 | GSE14333 | NA | included |
| GSM358478 | GSE14333 | NA | included |
| GSM358479 | GSE14333 | NA | included |
| GSM358480 | GSE14333 | NA | included |
| GSM358481 | GSE14333 | NA | included |
| GSM358482 | GSE14333 | NA | rejected |
| GSM358483 | GSE14333 | NA | included |
| GSM358484 | GSE14333 | NA | included |
| GSM358485 | GSE14333 | NA | included |
| GSM358486 | GSE14333 | NA | included |
| GSM358487 | GSE14333 | NA | included |
| GSM358488 | GSE14333 | NA | included |
| GSM358489 | GSE14333 | NA | included |
| GSM358490 | GSE14333 | NA | included |
| GSM358491 | GSE14333 | NA | included |
| GSM358492 | GSE14333 | NA | included |
| GSM358493 | GSE14333 | NA | included |
| GSM358494 | GSE14333 | NA | included |
| GSM358495 | GSE14333 | NA | included |
| GSM358496 | GSE14333 | NA | rejected |
| GSM358497 | GSE14333 | NA | included |
| GSM358498 | GSE14333 | NA | included |
| GSM358499 | GSE14333 | NA | included |
| GSM358500 | GSE14333 | NA | included |
| GSM358501 | GSE14333 | NA | included |
| GSM358502 | GSE14333 | NA | included |
| GSM358503 | GSE14333 | NA | included |
| GSM358504 | GSE14333 | NA | rejected |
| GSM358505 | GSE14333 | NA | included |
| GSM358506 | GSE14333 | NA | included |
| GSM358507 | GSE14333 | NA | included |
| GSM358508 | GSE14333 | NA | included |
| GSM358509 | GSE14333 | NA | included |
| GSM358510 | GSE14333 | NA | included |
| GSM358511 | GSE14333 | NA | rejected |
| GSM358512 | GSE14333 | NA | included |
| GSM358513 | GSE14333 | NA | rejected |
| GSM358514 | GSE14333 | NA | included |
| GSM358515 | GSE14333 | NA | rejected |
| GSM358516 | GSE14333 | NA | included |
| GSM358517 | GSE14333 | NA | included |
| GSM358518 | GSE14333 | NA | included |
| GSM358519 | GSE14333 | NA | included |
| GSM358520 | GSE14333 | NA | included |
| GSM358521 | GSE14333 | NA | included |
| GSM358522 | GSE14333 | NA | included |
| GSM358523 | GSE14333 | NA | included |
| GSM358524 | GSE14333 | NA | included |
| GSM358525 | GSE14333 | NA | included |
| GSM358526 | GSE14333 | NA | included |
| GSM358527 | GSE14333 | NA | included |
| GSM358528 | GSE14333 | NA | included |
| GSM358529 | GSE14333 | NA | included |
| GSM358530 | GSE14333 | NA | included |
| GSM358531 | GSE14333 | NA | included |
| GSM358532 | GSE14333 | NA | included |
| GSM358533 | GSE14333 | NA | rejected |
| GSM358534 | GSE14333 | NA | included |
| GSM358535 | GSE14333 | NA | included |
| GSM358536 | GSE14333 | NA | included |
| GSM358537 | GSE14333 | NA | included |
| GSM358538 | GSE14333 | NA | included |
| GSM358539 | GSE14333 | NA | included |
| GSM358540 | GSE14333 | NA | included |
| GSM358541 | GSE14333 | NA | included |
| GSM358542 | GSE14333 | NA | included |
| GSM358543 | GSE14333 | NA | included |
| GSM358544 | GSE14333 | NA | included |
| GSM358545 | GSE14333 | NA | rejected |
| GSM358546 | GSE14333 | NA | rejected |
| GSM358547 | GSE14333 | NA | included |
| GSM358548 | GSE14333 | NA | included |
| GSM358549 | GSE14333 | NA | rejected |
| GSM358550 | GSE14333 | NA | included |
| GSM358551 | GSE14333 | NA | included |
| GSM358552 | GSE14333 | NA | rejected |
| GSM358553 | GSE14333 | NA | included |
| GSM358554 | GSE14333 | NA | included |
| GSM358555 | GSE14333 | NA | rejected |
| GSM358556 | GSE14333 | NA | included |
| GSM358557 | GSE14333 | NA | rejected |
| GSM358558 | GSE14333 | NA | rejected |
| GSM358559 | GSE14333 | NA | included |
| GSM358560 | GSE14333 | NA | included |
| GSM358561 | GSE14333 | NA | rejected |
| GSM358562 | GSE14333 | NA | included |
| GSM358563 | GSE14333 | NA | rejected |
| GSM358564 | GSE14333 | NA | included |
| GSM358565 | GSE14333 | NA | included |
| GSM358566 | GSE14333 | NA | included |
| GSM358567 | GSE14333 | NA | rejected |
| GSM358568 | GSE14333 | NA | rejected |
| GSM358569 | GSE14333 | NA | included |
| GSM358570 | GSE14333 | NA | included |
| GSM358571 | GSE14333 | NA | included |
| GSM358572 | GSE14333 | NA | included |
| GSM358573 | GSE14333 | NA | included |
| GSM358574 | GSE14333 | NA | included |
| GSM358575 | GSE14333 | NA | included |
| GSM358576 | GSE14333 | NA | included |
| GSM358577 | GSE14333 | NA | included |
| GSM358578 | GSE14333 | NA | rejected |
| GSM358579 | GSE14333 | NA | included |
| GSM358580 | GSE14333 | NA | included |
| GSM358581 | GSE14333 | NA | included |
| GSM358582 | GSE14333 | NA | included |
| GSM358583 | GSE14333 | NA | included |
| GSM358584 | GSE14333 | NA | rejected |
| GSM358585 | GSE14333 | NA | included |
| GSM358586 | GSE14333 | NA | included |
| GSM358587 | GSE14333 | NA | included |
| GSM358588 | GSE14333 | NA | included |
| GSM358589 | GSE14333 | NA | included |
| GSM358590 | GSE14333 | NA | included |
| GSM358591 | GSE14333 | NA | rejected |
| GSM358592 | GSE14333 | NA | included |
| GSM358593 | GSE14333 | NA | included |
| GSM358594 | GSE14333 | NA | included |
| GSM358595 | GSE14333 | NA | included |
| GSM358596 | GSE14333 | NA | included |
| GSM358597 | GSE14333 | NA | included |
| GSM358598 | GSE14333 | NA | included |
| GSM358599 | GSE14333 | NA | rejected |
| GSM358600 | GSE14333 | NA | rejected |
| GSM358601 | GSE14333 | NA | rejected |
| GSM358602 | GSE14333 | NA | included |
| GSM358603 | GSE14333 | NA | included |
| GSM358604 | GSE14333 | NA | included |
| GSM358605 | GSE14333 | NA | included |
| GSM358606 | GSE14333 | NA | included |
| GSM358607 | GSE14333 | NA | rejected |
| GSM358608 | GSE14333 | NA | included |
| GSM358609 | GSE14333 | NA | included |
| GSM358610 | GSE14333 | NA | included |
| GSM358611 | GSE14333 | NA | included |
| GSM358612 | GSE14333 | NA | included |
| GSM358613 | GSE14333 | NA | included |
| GSM358614 | GSE14333 | NA | included |
| GSM358615 | GSE14333 | NA | included |
| GSM358616 | GSE14333 | NA | rejected |
| GSM358617 | GSE14333 | NA | rejected |
| GSM358618 | GSE14333 | NA | included |
| GSM358619 | GSE14333 | NA | included |
| GSM358620 | GSE14333 | NA | included |
| GSM358621 | GSE14333 | NA | included |
| GSM358622 | GSE14333 | NA | included |
| GSM358623 | GSE14333 | NA | included |
| GSM358624 | GSE14333 | NA | included |
| GSM358625 | GSE14333 | NA | included |
| GSM358626 | GSE14333 | NA | included |
| GSM358627 | GSE14333 | NA | included |
| GSM358628 | GSE14333 | NA | included |
| GSM358629 | GSE14333 | NA | included |
| GSM358630 | GSE14333 | NA | included |
| GSM437093 | GSE17536 | NA | rejected |
| GSM437094 | GSE17536 | NA | included |
| GSM437095 | GSE17536 | NA | included |
| GSM437096 | GSE17536 | NA | included |
| GSM437097 | GSE17536 | NA | included |
| GSM437098 | GSE17536 | NA | included |
| GSM437099 | GSE17536 | NA | included |
| GSM437100 | GSE17536 | NA | rejected |
| GSM437101 | GSE17536 | NA | included |
| GSM437102 | GSE17536 | NA | included |
| GSM437103 | GSE17536 | NA | rejected |
| GSM437104 | GSE17536 | NA | included |
| GSM437105 | GSE17536 | NA | included |
| GSM437106 | GSE17536 | NA | included |
| GSM437107 | GSE17536 | NA | included |
| GSM437108 | GSE17536 | NA | included |
| GSM437109 | GSE17536 | NA | included |
| GSM437110 | GSE17536 | NA | included |
| GSM437111 | GSE17536 | NA | included |
| GSM437112 | GSE17536 | NA | included |
| GSM437113 | GSE17536 | NA | included |
| GSM437114 | GSE17536 | NA | included |
| GSM437115 | GSE17536 | NA | included |
| GSM437116 | GSE17536 | NA | included |
| GSM437117 | GSE17536 | NA | included |
| GSM437118 | GSE17536 | NA | included |
| GSM437119 | GSE17536 | NA | included |
| GSM437120 | GSE17536 | NA | rejected |
| GSM437121 | GSE17536 | NA | rejected |
| GSM437122 | GSE17536 | NA | included |
| GSM437123 | GSE17536 | NA | rejected |
| GSM437124 | GSE17536 | NA | included |
| GSM437125 | GSE17536 | NA | included |
| GSM437126 | GSE17536 | NA | included |
| GSM437127 | GSE17536 | NA | rejected |
| GSM437128 | GSE17536 | NA | included |
| GSM437129 | GSE17536 | NA | included |
| GSM437130 | GSE17536 | NA | included |
| GSM437131 | GSE17536 | NA | rejected |
| GSM437132 | GSE17536 | NA | included |
| GSM437133 | GSE17536 | NA | included |
| GSM437134 | GSE17536 | NA | rejected |
| GSM437135 | GSE17536 | NA | included |
| GSM437136 | GSE17536 | NA | included |
| GSM437137 | GSE17536 | NA | included |
| GSM437138 | GSE17536 | NA | included |
| GSM437139 | GSE17536 | NA | included |
| GSM437140 | GSE17536 | NA | included |
| GSM437141 | GSE17536 | NA | included |
| GSM437142 | GSE17536 | NA | included |
| GSM437143 | GSE17536 | NA | included |
| GSM437144 | GSE17536 | NA | rejected |
| GSM437145 | GSE17536 | NA | included |
| GSM437146 | GSE17536 | NA | included |
| GSM437147 | GSE17536 | NA | included |
| GSM437148 | GSE17536 | NA | included |
| GSM437149 | GSE17536 | NA | rejected |
| GSM437150 | GSE17536 | NA | included |
| GSM437151 | GSE17536 | NA | included |
| GSM437152 | GSE17536 | NA | included |
| GSM437153 | GSE17536 | NA | included |
| GSM437154 | GSE17536 | NA | rejected |
| GSM437155 | GSE17536 | NA | included |
| GSM437156 | GSE17536 | NA | included |
| GSM437157 | GSE17536 | NA | included |
| GSM437158 | GSE17536 | NA | included |
| GSM437159 | GSE17536 | NA | rejected |
| GSM437160 | GSE17536 | NA | included |
| GSM437161 | GSE17536 | NA | included |
| GSM437162 | GSE17536 | NA | rejected |
| GSM437163 | GSE17536 | NA | rejected |
| GSM437164 | GSE17536 | NA | included |
| GSM437165 | GSE17536 | NA | included |
| GSM437166 | GSE17536 | NA | rejected |
| GSM437167 | GSE17536 | NA | included |
| GSM437168 | GSE17536 | NA | included |
| GSM437169 | GSE17536 | NA | included |
| GSM437170 | GSE17536 | NA | included |
| GSM437171 | GSE17536 | NA | included |
| GSM437172 | GSE17536 | NA | included |
| GSM437173 | GSE17536 | NA | included |
| GSM437174 | GSE17536 | NA | included |
| GSM437175 | GSE17536 | NA | included |
| GSM437176 | GSE17536 | NA | included |
| GSM437177 | GSE17536 | NA | included |
| GSM437178 | GSE17536 | NA | included |
| GSM437179 | GSE17536 | NA | included |
| GSM437180 | GSE17536 | NA | included |
| GSM437181 | GSE17536 | NA | included |
| GSM437182 | GSE17536 | NA | included |
| GSM437183 | GSE17536 | NA | included |
| GSM437184 | GSE17536 | NA | included |
| GSM437185 | GSE17536 | NA | included |
| GSM437186 | GSE17536 | NA | included |
| GSM437187 | GSE17536 | NA | rejected |
| GSM437188 | GSE17536 | NA | included |
| GSM437189 | GSE17536 | NA | included |
| GSM437190 | GSE17536 | NA | included |
| GSM437191 | GSE17536 | NA | rejected |
| GSM437192 | GSE17536 | NA | included |
| GSM437193 | GSE17536 | NA | included |
| GSM437194 | GSE17536 | NA | included |
| GSM437195 | GSE17536 | NA | rejected |
| GSM437196 | GSE17536 | NA | included |
| GSM437197 | GSE17536 | NA | included |
| GSM437198 | GSE17536 | NA | included |
| GSM437199 | GSE17536 | NA | included |
| GSM437200 | GSE17536 | NA | included |
| GSM437201 | GSE17536 | NA | included |
| GSM437202 | GSE17536 | NA | included |
| GSM437203 | GSE17536 | NA | included |
| GSM437204 | GSE17536 | NA | included |
| GSM437205 | GSE17536 | NA | rejected |
| GSM437206 | GSE17536 | NA | included |
| GSM437207 | GSE17536 | NA | included |
| GSM437208 | GSE17536 | NA | included |
| GSM437209 | GSE17536 | NA | included |
| GSM437210 | GSE17536 | NA | included |
| GSM437211 | GSE17536 | NA | included |
| GSM437212 | GSE17536 | NA | included |
| GSM437213 | GSE17536 | NA | included |
| GSM437214 | GSE17536 | NA | rejected |
| GSM437215 | GSE17536 | NA | rejected |
| GSM437216 | GSE17536 | NA | rejected |
| GSM437217 | GSE17536 | NA | included |
| GSM437218 | GSE17536 | NA | rejected |
| GSM437219 | GSE17536 | NA | included |
| GSM437220 | GSE17536 | NA | included |
| GSM437221 | GSE17536 | NA | included |
| GSM437222 | GSE17536 | NA | included |
| GSM437223 | GSE17536 | NA | included |
| GSM437224 | GSE17536 | NA | included |
| GSM437225 | GSE17536 | NA | included |
| GSM437226 | GSE17536 | NA | included |
| GSM437227 | GSE17536 | NA | included |
| GSM437228 | GSE17536 | NA | included |
| GSM437229 | GSE17536 | NA | included |
| GSM437230 | GSE17536 | NA | included |
| GSM437231 | GSE17536 | NA | rejected |
| GSM437232 | GSE17536 | NA | rejected |
| GSM437233 | GSE17536 | NA | rejected |
| GSM437234 | GSE17536 | NA | included |
| GSM437235 | GSE17536 | NA | included |
| GSM437236 | GSE17536 | NA | included |
| GSM437237 | GSE17536 | NA | included |
| GSM437238 | GSE17536 | NA | included |
| GSM437239 | GSE17536 | NA | included |
| GSM437240 | GSE17536 | NA | included |
| GSM437241 | GSE17536 | NA | included |
| GSM437242 | GSE17536 | NA | included |
| GSM437243 | GSE17536 | NA | included |
| GSM437244 | GSE17536 | NA | included |
| GSM437245 | GSE17536 | NA | included |
| GSM437246 | GSE17536 | NA | included |
| GSM437247 | GSE17536 | NA | included |
| GSM437248 | GSE17536 | NA | included |
| GSM437249 | GSE17536 | NA | included |
| GSM437250 | GSE17536 | NA | included |
| GSM437251 | GSE17536 | NA | rejected |
| GSM437252 | GSE17536 | NA | included |
| GSM437253 | GSE17536 | NA | included |
| GSM437254 | GSE17536 | NA | included |
| GSM437255 | GSE17536 | NA | included |
| GSM437256 | GSE17536 | NA | included |
| GSM437257 | GSE17536 | NA | included |
| GSM437258 | GSE17536 | NA | included |
| GSM437259 | GSE17536 | NA | rejected |
| GSM437260 | GSE17536 | NA | rejected |
| GSM437261 | GSE17536 | NA | included |
| GSM437262 | GSE17536 | NA | included |
| GSM437263 | GSE17536 | NA | included |
| GSM437264 | GSE17536 | NA | included |
| GSM437265 | GSE17536 | NA | included |
| GSM437266 | GSE17536 | NA | rejected |
| GSM437267 | GSE17536 | NA | included |
| GSM437268 | GSE17536 | NA | included |
| GSM437269 | GSE17536 | NA | included |
| GSM437270 | GSE17537 | NA | included |
| GSM437271 | GSE17537 | NA | included |
| GSM437272 | GSE17537 | NA | included |
| GSM437273 | GSE17537 | NA | included |
| GSM437274 | GSE17537 | NA | rejected |
| GSM437275 | GSE17537 | NA | included |
| GSM437276 | GSE17537 | NA | included |
| GSM437277 | GSE17537 | NA | included |
| GSM437278 | GSE17537 | NA | included |
| GSM437279 | GSE17537 | NA | included |
| GSM437280 | GSE17537 | NA | included |
| GSM437281 | GSE17537 | NA | included |
| GSM437282 | GSE17537 | NA | included |
| GSM437283 | GSE17537 | NA | rejected |
| GSM437284 | GSE17537 | NA | included |
| GSM437285 | GSE17537 | NA | included |
| GSM437286 | GSE17537 | NA | included |
| GSM437287 | GSE17537 | NA | included |
| GSM437288 | GSE17537 | NA | included |
| GSM437289 | GSE17537 | NA | included |
| GSM437290 | GSE17537 | NA | included |
| GSM437291 | GSE17537 | NA | included |
| GSM437292 | GSE17537 | NA | included |
| GSM437293 | GSE17537 | NA | included |
| GSM437294 | GSE17537 | NA | included |
| GSM437295 | GSE17537 | NA | rejected |
| GSM437296 | GSE17537 | NA | included |
| GSM437297 | GSE17537 | NA | included |
| GSM437298 | GSE17537 | NA | included |
| GSM437299 | GSE17537 | NA | rejected |
| GSM437300 | GSE17537 | NA | included |
| GSM437301 | GSE17537 | NA | included |
| GSM437302 | GSE17537 | NA | included |
| GSM437303 | GSE17537 | NA | included |
| GSM437304 | GSE17537 | NA | rejected |
| GSM437305 | GSE17537 | NA | included |
| GSM437306 | GSE17537 | NA | rejected |
| GSM437307 | GSE17537 | NA | included |
| GSM437308 | GSE17537 | NA | included |
| GSM437309 | GSE17537 | NA | included |
| GSM437310 | GSE17537 | NA | rejected |
| GSM437311 | GSE17537 | IIA | included |
| GSM437312 | GSE17537 | NA | included |
| GSM437313 | GSE17537 | NA | included |
| GSM437314 | GSE17537 | NA | included |
| GSM437315 | GSE17537 | NA | included |
| GSM437316 | GSE17537 | NA | rejected |
| GSM437317 | GSE17537 | NA | included |
| GSM437318 | GSE17537 | NA | included |
| GSM437319 | GSE17537 | NA | included |
| GSM437320 | GSE17537 | NA | rejected |
| GSM437321 | GSE17537 | NA | included |
| GSM437322 | GSE17537 | NA | included |
| GSM437323 | GSE17537 | NA | rejected |
| GSM437324 | GSE17537 | NA | included |
| GSM452629 | GSE18105 | IIIC | rejected |
| GSM452630 | GSE18105 | NA | rejected |
| GSM452631 | GSE18105 | NA | rejected |
| GSM452632 | GSE18105 | NA | rejected |
| GSM452633 | GSE18105 | NA | rejected |
| GSM452634 | GSE18105 | NA | rejected |
| GSM452635 | GSE18105 | NA | rejected |
| GSM452636 | GSE18105 | NA | rejected |
| GSM452637 | GSE18105 | IIA | rejected |
| GSM452638 | GSE18105 | NA | rejected |
| GSM452639 | GSE18105 | NA | rejected |
| GSM452640 | GSE18105 | I | rejected |
| GSM452641 | GSE18105 | NA | rejected |
| GSM452642 | GSE18105 | NA | rejected |
| GSM452643 | GSE18105 | NA | rejected |
| GSM452644 | GSE18105 | NA | rejected |
| GSM452645 | GSE18105 | NA | rejected |
| GSM496015 | GSE19860 | NA | included |
| GSM496016 | GSE19860 | NA | rejected |
| GSM496017 | GSE19860 | NA | included |
| GSM496018 | GSE19860 | NA | included |
| GSM496019 | GSE19860 | NA | rejected |
| GSM496020 | GSE19860 | NA | included |
| GSM496021 | GSE19860 | NA | rejected |
| GSM496022 | GSE19860 | NA | included |
| GSM496023 | GSE19860 | NA | included |
| GSM496024 | GSE19860 | NA | included |
| GSM496025 | GSE19860 | NA | included |
| GSM496026 | GSE19860 | NA | included |
| GSM496027 | GSE19860 | NA | included |
| GSM496028 | GSE19860 | NA | included |
| GSM496029 | GSE19860 | NA | rejected |
| GSM496030 | GSE19860 | NA | included |
| GSM496031 | GSE19860 | NA | rejected |
| GSM496032 | GSE19860 | NA | included |
| GSM496033 | GSE19860 | NA | included |
| GSM496034 | GSE19860 | NA | included |
| GSM496035 | GSE19860 | NA | rejected |
| GSM496036 | GSE19860 | NA | included |
| GSM496037 | GSE19860 | NA | included |
| GSM496038 | GSE19860 | NA | included |
| GSM496039 | GSE19860 | NA | included |
| GSM496040 | GSE19860 | NA | included |
| GSM496041 | GSE19860 | NA | included |
| GSM496042 | GSE19860 | NA | rejected |
| GSM496043 | GSE19860 | NA | rejected |
| GSM710801 | GSE28702 | NA | included |
| GSM710802 | GSE28702 | NA | included |
| GSM710803 | GSE28702 | NA | included |
| GSM710804 | GSE28702 | IIIC | rejected |
| GSM710805 | GSE28702 | NA | rejected |
| GSM710806 | GSE28702 | NA | rejected |
| GSM710807 | GSE28702 | NA | included |
| GSM710808 | GSE28702 | NA | included |
| GSM710809 | GSE28702 | NA | included |
| GSM710810 | GSE28702 | NA | included |
| GSM710811 | GSE28702 | NA | rejected |
| GSM710812 | GSE28702 | NA | included |
| GSM710813 | GSE28702 | NA | included |
| GSM710814 | GSE28702 | NA | included |
| GSM710815 | GSE28702 | NA | included |
| GSM710816 | GSE28702 | NA | included |
| GSM710817 | GSE28702 | NA | included |
| GSM710818 | GSE28702 | NA | included |
| GSM710819 | GSE28702 | NA | included |
| GSM710820 | GSE28702 | NA | included |
| GSM710821 | GSE28702 | NA | included |
| GSM710822 | GSE28702 | NA | included |
| GSM710823 | GSE28702 | NA | included |
| GSM710824 | GSE28702 | NA | included |
| GSM710825 | GSE28702 | NA | included |
| GSM710826 | GSE28702 | NA | included |
| GSM710827 | GSE28702 | NA | included |
| GSM710828 | GSE28702 | NA | included |
| GSM710829 | GSE28702 | NA | included |
| GSM710830 | GSE28702 | NA | included |
| GSM710831 | GSE28702 | NA | included |
| GSM710832 | GSE28702 | NA | included |
| GSM710833 | GSE28702 | NA | included |
| GSM710834 | GSE28702 | NA | included |
| GSM710835 | GSE28702 | NA | rejected |
| GSM710836 | GSE28702 | NA | included |
| GSM710837 | GSE28702 | NA | included |
| GSM710839 | GSE28702 | NA | included |
| GSM710841 | GSE28702 | NA | included |
| GSM710843 | GSE28702 | NA | included |
| GSM710845 | GSE28702 | NA | included |
| GSM710846 | GSE28702 | NA | included |
| GSM710849 | GSE28702 | NA | included |
| GSM710853 | GSE28702 | NA | included |
| GSM710855 | GSE28702 | NA | included |
| GSM710858 | GSE28702 | NA | included |
| GSM710860 | GSE28702 | NA | included |
| GSM710862 | GSE28702 | NA | included |
| GSM710863 | GSE28702 | NA | included |
| GSM710865 | GSE28702 | NA | rejected |
| GSM710867 | GSE28702 | NA | rejected |
| GSM710869 | GSE28702 | NA | rejected |
| GSM710871 | GSE28702 | NA | rejected |
| GSM710873 | GSE28702 | NA | included |
| GSM710875 | GSE28702 | NA | included |
| GSM710877 | GSE28702 | NA | included |
| GSM710879 | GSE28702 | NA | included |
| GSM710881 | GSE28702 | NA | included |
| GSM710883 | GSE28702 | NA | included |
| GSM710885 | GSE28702 | NA | rejected |
| GSM710886 | GSE28702 | NA | rejected |
| GSM710888 | GSE28702 | NA | rejected |
| GSM710890 | GSE28702 | NA | rejected |
| GSM710892 | GSE28702 | NA | rejected |
| GSM710894 | GSE28702 | NA | rejected |
| GSM710896 | GSE28702 | NA | included |
| GSM710898 | GSE28702 | NA | rejected |
| GSM710900 | GSE28702 | NA | rejected |
| GSM710902 | GSE28702 | NA | included |
| GSM710905 | GSE28702 | NA | included |
| GSM710906 | GSE28702 | NA | rejected |
| GSM710908 | GSE28702 | NA | included |
| GSM710911 | GSE28702 | NA | included |
| GSM710913 | GSE28702 | NA | included |
| GSM710915 | GSE28702 | NA | included |
| GSM710916 | GSE28702 | NA | included |
| GSM710918 | GSE28702 | NA | included |
| GSM710920 | GSE28702 | NA | rejected |
| GSM710922 | GSE28702 | NA | rejected |
| GSM710924 | GSE28702 | NA | included |
| GSM710926 | GSE28702 | NA | included |
| GSM710928 | GSE28702 | NA | rejected |
| GSM710930 | GSE28702 | NA | rejected |
| GSM820048 | GSE33113 | NA | rejected |
| GSM820049 | GSE33113 | NA | included |
| GSM820050 | GSE33113 | NA | included |
| GSM820051 | GSE33113 | NA | included |
| GSM820052 | GSE33113 | NA | included |
| GSM820053 | GSE33113 | NA | included |
| GSM820054 | GSE33113 | NA | rejected |
| GSM820055 | GSE33113 | NA | included |
| GSM820056 | GSE33113 | NA | included |
| GSM820057 | GSE33113 | NA | included |
| GSM820058 | GSE33113 | NA | rejected |
| GSM820059 | GSE33113 | NA | included |
| GSM820060 | GSE33113 | NA | included |
| GSM820061 | GSE33113 | NA | included |
| GSM820062 | GSE33113 | NA | included |
| GSM820063 | GSE33113 | NA | included |
| GSM820064 | GSE33113 | NA | rejected |
| GSM820065 | GSE33113 | NA | rejected |
| GSM820066 | GSE33113 | NA | included |
| GSM820067 | GSE33113 | NA | included |
| GSM820068 | GSE33113 | NA | included |
| GSM820069 | GSE33113 | NA | included |
| GSM820070 | GSE33113 | NA | included |
| GSM820071 | GSE33113 | NA | included |
| GSM820072 | GSE33113 | NA | included |
| GSM820073 | GSE33113 | NA | included |
| GSM820074 | GSE33113 | NA | included |
| GSM820075 | GSE33113 | NA | included |
| GSM820076 | GSE33113 | NA | included |
| GSM820077 | GSE33113 | NA | included |
| GSM820078 | GSE33113 | NA | included |
| GSM820079 | GSE33113 | NA | included |
| GSM820080 | GSE33113 | NA | included |
| GSM820081 | GSE33113 | NA | included |
| GSM820082 | GSE33113 | NA | included |
| GSM820083 | GSE33113 | NA | included |
| GSM820084 | GSE33113 | NA | included |
| GSM820085 | GSE33113 | NA | rejected |
| GSM820086 | GSE33113 | NA | included |
| GSM820087 | GSE33113 | NA | rejected |
| GSM820088 | GSE33113 | NA | included |
| GSM820089 | GSE33113 | NA | included |
| GSM820090 | GSE33113 | NA | included |
| GSM820091 | GSE33113 | NA | rejected |
| GSM820092 | GSE33113 | NA | included |
| GSM820093 | GSE33113 | NA | included |
| GSM820094 | GSE33113 | NA | included |
| GSM820095 | GSE33113 | NA | included |
| GSM820096 | GSE33113 | NA | included |
| GSM820097 | GSE33113 | NA | rejected |
| GSM820098 | GSE33113 | NA | included |
| GSM820099 | GSE33113 | NA | included |
| GSM820100 | GSE33113 | NA | included |
| GSM820101 | GSE33113 | NA | included |
| GSM820102 | GSE33113 | NA | included |
| GSM820103 | GSE33113 | NA | included |
| GSM820104 | GSE33113 | NA | included |
| GSM820105 | GSE33113 | NA | included |
| GSM820106 | GSE33113 | NA | included |
| GSM820107 | GSE33113 | NA | included |
| GSM820108 | GSE33113 | NA | included |
| GSM820109 | GSE33113 | NA | included |
| GSM820110 | GSE33113 | NA | included |
| GSM820111 | GSE33113 | NA | included |
| GSM820112 | GSE33113 | NA | included |
| GSM820113 | GSE33113 | NA | included |
| GSM820114 | GSE33113 | NA | rejected |
| GSM820115 | GSE33113 | NA | included |
| GSM820116 | GSE33113 | NA | rejected |
| GSM820117 | GSE33113 | NA | rejected |
| GSM820118 | GSE33113 | NA | included |
| GSM820119 | GSE33113 | NA | rejected |
| GSM820120 | GSE33113 | NA | included |
| GSM820121 | GSE33113 | NA | included |
| GSM820122 | GSE33113 | NA | included |
| GSM820123 | GSE33113 | NA | rejected |
| GSM820124 | GSE33113 | NA | rejected |
| GSM820125 | GSE33113 | NA | rejected |
| GSM820126 | GSE33113 | NA | included |
| GSM820127 | GSE33113 | NA | included |
| GSM820128 | GSE33113 | NA | included |
| GSM820129 | GSE33113 | NA | included |
| GSM820130 | GSE33113 | NA | included |
| GSM820131 | GSE33113 | NA | included |
| GSM820132 | GSE33113 | NA | included |
| GSM820133 | GSE33113 | NA | included |
| GSM820134 | GSE33113 | NA | included |
| GSM820135 | GSE33113 | NA | included |
| GSM820136 | GSE33113 | NA | rejected |
| GSM820137 | GSE33113 | NA | included |
| GSM877126 | GSE35896 | NA | rejected |
| GSM877127 | GSE35896 | NA | rejected |
| GSM877128 | GSE35896 | NA | included |
| GSM877129 | GSE35896 | NA | rejected |
| GSM877130 | GSE35896 | NA | included |
| GSM877131 | GSE35896 | NA | rejected |
| GSM877132 | GSE35896 | IIA | rejected |
| GSM877133 | GSE35896 | NA | included |
| GSM877134 | GSE35896 | NA | rejected |
| GSM877135 | GSE35896 | NA | included |
| GSM877136 | GSE35896 | NA | included |
| GSM877137 | GSE35896 | NA | rejected |
| GSM877138 | GSE35896 | NA | included |
| GSM877139 | GSE35896 | NA | included |
| GSM877140 | GSE35896 | NA | included |
| GSM877141 | GSE35896 | NA | rejected |
| GSM877142 | GSE35896 | NA | rejected |
| GSM877143 | GSE35896 | NA | included |
| GSM877144 | GSE35896 | NA | included |
| GSM877145 | GSE35896 | IIB/IIC | rejected |
| GSM877146 | GSE35896 | NA | included |
| GSM877147 | GSE35896 | NA | rejected |
| GSM877148 | GSE35896 | NA | included |
| GSM877149 | GSE35896 | NA | rejected |
| GSM877150 | GSE35896 | NA | included |
| GSM877151 | GSE35896 | NA | rejected |
| GSM877152 | GSE35896 | NA | included |
| GSM877153 | GSE35896 | NA | included |
| GSM877154 | GSE35896 | NA | included |
| GSM877155 | GSE35896 | NA | included |
| GSM877156 | GSE35896 | I | rejected |
| GSM877157 | GSE35896 | NA | rejected |
| GSM877158 | GSE35896 | NA | included |
| GSM877159 | GSE35896 | NA | included |
| GSM877160 | GSE35896 | NA | included |
| GSM877161 | GSE35896 | NA | included |
| GSM877162 | GSE35896 | NA | rejected |
| GSM877163 | GSE35896 | NA | rejected |
| GSM877164 | GSE35896 | NA | included |
| GSM877165 | GSE35896 | NA | rejected |
| GSM877166 | GSE35896 | NA | included |
| GSM877167 | GSE35896 | NA | rejected |
| GSM877168 | GSE35896 | NA | included |
| GSM877169 | GSE35896 | NA | included |
| GSM877170 | GSE35896 | NA | rejected |
| GSM877171 | GSE35896 | NA | included |
| GSM877173 | GSE35896 | NA | rejected |
| GSM877174 | GSE35896 | NA | rejected |
| GSM877175 | GSE35896 | NA | included |
| GSM877176 | GSE35896 | NA | rejected |
| GSM877177 | GSE35896 | NA | included |
| GSM877178 | GSE35896 | NA | included |
| GSM877179 | GSE35896 | NA | rejected |
| GSM877180 | GSE35896 | NA | included |
| GSM877181 | GSE35896 | NA | included |
| GSM877182 | GSE35896 | NA | rejected |
| GSM877183 | GSE35896 | NA | included |
| GSM877184 | GSE35896 | NA | included |
| GSM877185 | GSE35896 | NA | included |
| GSM877186 | GSE35896 | NA | included |
| GSM877187 | GSE35896 | NA | included |
| GSM877188 | GSE35896 | IIB/IIC | rejected |
| GSM916743 | GSE37364 | NA | rejected |
| GSM916744 | GSE37364 | NA | included |
| GSM916745 | GSE37364 | NA | included |
| GSM916746 | GSE37364 | NA | included |
| GSM916747 | GSE37364 | NA | included |
| GSM916748 | GSE37364 | NA | included |
| GSM916749 | GSE37364 | NA | included |
| GSM916750 | GSE37364 | NA | included |
| GSM916751 | GSE37364 | NA | included |
| GSM916752 | GSE37364 | NA | included |
| GSM916753 | GSE37364 | NA | included |
| GSM916754 | GSE37364 | NA | included |
| GSM916755 | GSE37364 | NA | included |
| GSM916756 | GSE37364 | NA | included |
| GSM916757 | GSE37364 | NA | included |
| GSM916758 | GSE37364 | NA | included |
| GSM916759 | GSE37364 | NA | included |
| GSM916760 | GSE37364 | NA | included |
| GSM916761 | GSE37364 | NA | included |
| GSM916762 | GSE37364 | NA | included |
| GSM916763 | GSE37364 | NA | included |
| GSM916764 | GSE37364 | NA | included |
| GSM916765 | GSE37364 | NA | included |
| GSM916766 | GSE37364 | NA | included |
| GSM916767 | GSE37364 | NA | included |
| GSM916768 | GSE37364 | NA | included |
| GSM916769 | GSE37364 | NA | included |
| GSM916770 | GSE37364 | NA | included |
| GSM916771 | GSE37364 | NA | included |
| GSM916772 | GSE37364 | NA | included |
| GSM916773 | GSE37364 | NA | included |
| GSM916774 | GSE37364 | NA | included |
| GSM916775 | GSE37364 | NA | included |
| GSM916776 | GSE37364 | NA | included |
| GSM916777 | GSE37364 | NA | included |
| GSM916778 | GSE37364 | NA | included |
| GSM916779 | GSE37364 | NA | included |
| GSM916780 | GSE37364 | NA | included |
| GSM971957 | GSE39582 | IV | included |
| GSM971958 | GSE39582 | IV | included |
| GSM971959 | GSE39582 | IIB/IIC | included |
| GSM971960 | GSE39582 | I | included |
| GSM971961 | GSE39582 | IV | included |
| GSM971962 | GSE39582 | IIIC | included |
| GSM971963 | GSE39582 | IIA | included |
| GSM971964 | GSE39582 | IIIB | included |
| GSM971965 | GSE39582 | IIA | rejected |
| GSM971966 | GSE39582 | IIIB | included |
| GSM971967 | GSE39582 | IIA | included |
| GSM971968 | GSE39582 | IIB/IIC | included |
| GSM971969 | GSE39582 | I | rejected |
| GSM971970 | GSE39582 | I | included |
| GSM971971 | GSE39582 | IV | included |
| GSM971972 | GSE39582 | I | included |
| GSM971973 | GSE39582 | IIA | included |
| GSM971974 | GSE39582 | IV | included |
| GSM971975 | GSE39582 | IIA | included |
| GSM971976 | GSE39582 | IIB/IIC | included |
| GSM971977 | GSE39582 | IIIC | included |
| GSM971978 | GSE39582 | I | included |
| GSM971979 | GSE39582 | IIA | included |
| GSM971980 | GSE39582 | IIIC | rejected |
| GSM971981 | GSE39582 | IIA | included |
| GSM971982 | GSE39582 | IIIC | included |
| GSM971983 | GSE39582 | IIIB | included |
| GSM971984 | GSE39582 | IIB/IIC | included |
| GSM971985 | GSE39582 | I | included |
| GSM971986 | GSE39582 | IIA | included |
| GSM971987 | GSE39582 | IIA | rejected |
| GSM971988 | GSE39582 | IV | included |
| GSM971989 | GSE39582 | I | included |
| GSM971990 | GSE39582 | I | rejected |
| GSM971991 | GSE39582 | IIA | included |
| GSM971992 | GSE39582 | IIB/IIC | included |
| GSM971993 | GSE39582 | IIA | included |
| GSM971994 | GSE39582 | IIA | included |
| GSM971995 | GSE39582 | IIB/IIC | rejected |
| GSM971996 | GSE39582 | IIIB | included |
| GSM971997 | GSE39582 | IIIC | rejected |
| GSM971998 | GSE39582 | IIIB | rejected |
| GSM971999 | GSE39582 | IIIB | rejected |
| GSM972000 | GSE39582 | IIIC | included |
| GSM972001 | GSE39582 | IIIA | included |
| GSM972002 | GSE39582 | IIIC | included |
| GSM972003 | GSE39582 | IV | included |
| GSM972004 | GSE39582 | IV | included |
| GSM972005 | GSE39582 | IV | included |
| GSM972006 | GSE39582 | IV | rejected |
| GSM972007 | GSE39582 | IV | included |
| GSM972008 | GSE39582 | IV | included |
| GSM972009 | GSE39582 | IV | rejected |
| GSM972010 | GSE39582 | IV | included |
| GSM972011 | GSE39582 | IV | included |
| GSM972012 | GSE39582 | IV | included |
| GSM972013 | GSE39582 | IV | included |
| GSM972014 | GSE39582 | IV | included |
| GSM972015 | GSE39582 | IIA | included |
| GSM972016 | GSE39582 | IIA | rejected |
| GSM972017 | GSE39582 | IIIC | included |
| GSM972018 | GSE39582 | IIIB | included |
| GSM972019 | GSE39582 | NA | included |
| GSM972020 | GSE39582 | IIIC | rejected |
| GSM972021 | GSE39582 | IIIC | included |
| GSM972022 | GSE39582 | IIIC | included |
| GSM972023 | GSE39582 | IIIB | included |
| GSM972024 | GSE39582 | IIIB | included |
| GSM972025 | GSE39582 | IIIC | included |
| GSM972026 | GSE39582 | IIIC | included |
| GSM972027 | GSE39582 | IIIC | included |
| GSM972028 | GSE39582 | IIIC | rejected |
| GSM972029 | GSE39582 | IIIC | rejected |
| GSM972030 | GSE39582 | NA | rejected |
| GSM972031 | GSE39582 | NA | rejected |
| GSM972032 | GSE39582 | IIA | included |
| GSM972033 | GSE39582 | IIA | rejected |
| GSM972034 | GSE39582 | NA | rejected |
| GSM972035 | GSE39582 | IIA | included |
| GSM972036 | GSE39582 | NA | rejected |
| GSM972037 | GSE39582 | IIB/IIC | included |
| GSM972038 | GSE39582 | IIA | included |
| GSM972039 | GSE39582 | NA | rejected |
| GSM972040 | GSE39582 | IIA | included |
| GSM972041 | GSE39582 | IIA | included |
| GSM972042 | GSE39582 | IIA | included |
| GSM972043 | GSE39582 | IV | included |
| GSM972044 | GSE39582 | IIB/IIC | included |
| GSM972045 | GSE39582 | IV | included |
| GSM972046 | GSE39582 | IIA | included |
| GSM972047 | GSE39582 | IIA | included |
| GSM972048 | GSE39582 | IV | included |
| GSM972049 | GSE39582 | IIA | included |
| GSM972050 | GSE39582 | IIA | included |
| GSM972051 | GSE39582 | IV | included |
| GSM972052 | GSE39582 | I | included |
| GSM972053 | GSE39582 | I | included |
| GSM972054 | GSE39582 | IIA | included |
| GSM972055 | GSE39582 | IIB/IIC | included |
| GSM972056 | GSE39582 | IIB/IIC | included |
| GSM972057 | GSE39582 | IIA | included |
| GSM972058 | GSE39582 | IIA | included |
| GSM972059 | GSE39582 | IIA | rejected |
| GSM972060 | GSE39582 | IIA | included |
| GSM972061 | GSE39582 | IIB/IIC | rejected |
| GSM972062 | GSE39582 | IIB/IIC | rejected |
| GSM972063 | GSE39582 | IIA | rejected |
| GSM972064 | GSE39582 | IV | included |
| GSM972065 | GSE39582 | IIA | rejected |
| GSM972066 | GSE39582 | IV | included |
| GSM972067 | GSE39582 | IIA | included |
| GSM972068 | GSE39582 | IIB/IIC | included |
| GSM972069 | GSE39582 | IIA | included |
| GSM972070 | GSE39582 | IIB/IIC | rejected |
| GSM972071 | GSE39582 | IIA | included |
| GSM972072 | GSE39582 | IIB/IIC | included |
| GSM972073 | GSE39582 | IIA | included |
| GSM972074 | GSE39582 | IIB/IIC | included |
| GSM972075 | GSE39582 | IIB/IIC | included |
| GSM972076 | GSE39582 | IIA | included |
| GSM972077 | GSE39582 | IIA | rejected |
| GSM972078 | GSE39582 | IIA | included |
| GSM972079 | GSE39582 | IIA | rejected |
| GSM972080 | GSE39582 | IIB/IIC | included |
| GSM972081 | GSE39582 | IIA | rejected |
| GSM972082 | GSE39582 | IIA | included |
| GSM972083 | GSE39582 | IIA | rejected |
| GSM972084 | GSE39582 | IIA | rejected |
| GSM972085 | GSE39582 | IIA | included |
| GSM972086 | GSE39582 | IIA | included |
| GSM972087 | GSE39582 | IIA | included |
| GSM972088 | GSE39582 | IIB/IIC | rejected |
| GSM972089 | GSE39582 | IIA | included |
| GSM972090 | GSE39582 | IIA | included |
| GSM972091 | GSE39582 | IIA | included |
| GSM972092 | GSE39582 | IIA | included |
| GSM972093 | GSE39582 | IIB/IIC | included |
| GSM972094 | GSE39582 | IIA | included |
| GSM972095 | GSE39582 | IIA | included |
| GSM972096 | GSE39582 | IIIA | included |
| GSM972097 | GSE39582 | IIIC | included |
| GSM972098 | GSE39582 | IIIC | included |
| GSM972099 | GSE39582 | IIIB | included |
| GSM972100 | GSE39582 | IIIC | included |
| GSM972101 | GSE39582 | IIIB | rejected |
| GSM972102 | GSE39582 | IIIB | rejected |
| GSM972103 | GSE39582 | IIIC | included |
| GSM972104 | GSE39582 | NA | included |
| GSM972105 | GSE39582 | NA | rejected |
| GSM972106 | GSE39582 | IIIB | included |
| GSM972107 | GSE39582 | IIIB | rejected |
| GSM972108 | GSE39582 | IIIC | included |
| GSM972109 | GSE39582 | IIIB | included |
| GSM972110 | GSE39582 | IIIC | included |
| GSM972111 | GSE39582 | IIIC | included |
| GSM972112 | GSE39582 | IIIC | included |
| GSM972113 | GSE39582 | IIIB | included |
| GSM972114 | GSE39582 | IIIB | included |
| GSM972115 | GSE39582 | NA | rejected |
| GSM972116 | GSE39582 | IIIC | rejected |
| GSM972117 | GSE39582 | IIIB | rejected |
| GSM972118 | GSE39582 | IIIC | included |
| GSM972119 | GSE39582 | IIIC | included |
| GSM972120 | GSE39582 | IIIB | included |
| GSM972121 | GSE39582 | IIIB | included |
| GSM972122 | GSE39582 | IIIC | included |
| GSM972123 | GSE39582 | IIA | included |
| GSM972124 | GSE39582 | IIA | rejected |
| GSM972125 | GSE39582 | IIA | rejected |
| GSM972126 | GSE39582 | IIA | included |
| GSM972127 | GSE39582 | NA | rejected |
| GSM972128 | GSE39582 | IIA | included |
| GSM972129 | GSE39582 | I | included |
| GSM972130 | GSE39582 | IIB/IIC | included |
| GSM972131 | GSE39582 | IIA | included |
| GSM972132 | GSE39582 | IIA | included |
| GSM972133 | GSE39582 | NA | rejected |
| GSM972134 | GSE39582 | IIIC | rejected |
| GSM972135 | GSE39582 | IIA | included |
| GSM972136 | GSE39582 | IIIB | rejected |
| GSM972137 | GSE39582 | IIA | included |
| GSM972138 | GSE39582 | IIA | included |
| GSM972139 | GSE39582 | IIIB | included |
| GSM972140 | GSE39582 | IIB/IIC | included |
| GSM972141 | GSE39582 | IIIC | included |
| GSM972142 | GSE39582 | IIIC | included |
| GSM972143 | GSE39582 | IIB/IIC | included |
| GSM972144 | GSE39582 | IIA | included |
| GSM972145 | GSE39582 | IIIC | rejected |
| GSM972146 | GSE39582 | IIIC | included |
| GSM972147 | GSE39582 | IIA | included |
| GSM972148 | GSE39582 | IIIB | included |
| GSM972149 | GSE39582 | IIA | included |
| GSM972150 | GSE39582 | IIIB | rejected |
| GSM972151 | GSE39582 | IIIB | included |
| GSM972152 | GSE39582 | IIIB | included |
| GSM972153 | GSE39582 | IIA | rejected |
| GSM972154 | GSE39582 | IIIC | included |
| GSM972155 | GSE39582 | IIIC | included |
| GSM972156 | GSE39582 | IIIB | rejected |
| GSM972157 | GSE39582 | IIIB | included |
| GSM972158 | GSE39582 | IIIC | included |
| GSM972159 | GSE39582 | IIIB | included |
| GSM972160 | GSE39582 | IIA | included |
| GSM972161 | GSE39582 | IIA | included |
| GSM972162 | GSE39582 | IIIB | included |
| GSM972163 | GSE39582 | IIB/IIC | rejected |
| GSM972164 | GSE39582 | NA | included |
| GSM972165 | GSE39582 | IIB/IIC | rejected |
| GSM972166 | GSE39582 | NA | rejected |
| GSM972167 | GSE39582 | IIA | included |
| GSM972168 | GSE39582 | NA | included |
| GSM972169 | GSE39582 | IIB/IIC | included |
| GSM972170 | GSE39582 | IIIB | included |
| GSM972171 | GSE39582 | IIIC | included |
| GSM972172 | GSE39582 | IIB/IIC | included |
| GSM972173 | GSE39582 | IIB/IIC | included |
| GSM972174 | GSE39582 | IIIB | included |
| GSM972175 | GSE39582 | IIIB | included |
| GSM972176 | GSE39582 | IIIC | included |
| GSM972177 | GSE39582 | NA | rejected |
| GSM972178 | GSE39582 | NA | included |
| GSM972179 | GSE39582 | NA | included |
| GSM972180 | GSE39582 | NA | included |
| GSM972181 | GSE39582 | NA | included |
| GSM972182 | GSE39582 | IIB/IIC | included |
| GSM972183 | GSE39582 | NA | included |
| GSM972184 | GSE39582 | NA | included |
| GSM972185 | GSE39582 | NA | included |
| GSM972186 | GSE39582 | NA | included |
| GSM972187 | GSE39582 | IIA | included |
| GSM972188 | GSE39582 | IIIC | included |
| GSM972189 | GSE39582 | NA | included |
| GSM972190 | GSE39582 | IIA | included |
| GSM972191 | GSE39582 | IIIB | included |
| GSM972192 | GSE39582 | IIA | rejected |
| GSM972193 | GSE39582 | IIIB | included |
| GSM972194 | GSE39582 | IIIC | included |
| GSM972195 | GSE39582 | IIA | included |
| GSM972196 | GSE39582 | IIA | included |
| GSM972197 | GSE39582 | IIA | included |
| GSM972198 | GSE39582 | IIIB | rejected |
| GSM972199 | GSE39582 | IIIC | included |
| GSM972200 | GSE39582 | IIA | rejected |
| GSM972201 | GSE39582 | IIIC | included |
| GSM972202 | GSE39582 | IIIB | rejected |
| GSM972203 | GSE39582 | IIIC | included |
| GSM972204 | GSE39582 | NA | included |
| GSM972205 | GSE39582 | NA | included |
| GSM972206 | GSE39582 | NA | rejected |
| GSM972207 | GSE39582 | IIIB | included |
| GSM972208 | GSE39582 | IV | included |
| GSM972209 | GSE39582 | IV | included |
| GSM972210 | GSE39582 | IV | included |
| GSM972211 | GSE39582 | IIIB | included |
| GSM972212 | GSE39582 | IV | included |
| GSM972213 | GSE39582 | IV | included |
| GSM972214 | GSE39582 | IV | included |
| GSM972215 | GSE39582 | IIIC | included |
| GSM972216 | GSE39582 | IV | included |
| GSM972217 | GSE39582 | IV | included |
| GSM972218 | GSE39582 | IV | included |
| GSM972219 | GSE39582 | IV | rejected |
| GSM972220 | GSE39582 | IIIB | included |
| GSM972221 | GSE39582 | IIA | included |
| GSM972222 | GSE39582 | IIA | included |
| GSM972223 | GSE39582 | IIA | included |
| GSM972224 | GSE39582 | IIIC | included |
| GSM972225 | GSE39582 | IIA | included |
| GSM972226 | GSE39582 | IIB/IIC | included |
| GSM972227 | GSE39582 | IV | included |
| GSM972228 | GSE39582 | IIA | included |
| GSM972229 | GSE39582 | IIA | included |
| GSM972230 | GSE39582 | IIIB | included |
| GSM972231 | GSE39582 | IV | included |
| GSM972232 | GSE39582 | IV | included |
| GSM972233 | GSE39582 | IIA | included |
| GSM972234 | GSE39582 | IV | included |
| GSM972235 | GSE39582 | IIIA | included |
| GSM972236 | GSE39582 | IV | included |
| GSM972237 | GSE39582 | I | included |
| GSM972238 | GSE39582 | IIIA | included |
| GSM972239 | GSE39582 | IIA | included |
| GSM972240 | GSE39582 | IV | included |
| GSM972241 | GSE39582 | IIA | included |
| GSM972242 | GSE39582 | I | included |
| GSM972243 | GSE39582 | IIA | included |
| GSM972244 | GSE39582 | I | rejected |
| GSM972245 | GSE39582 | IV | included |
| GSM972246 | GSE39582 | IIIB | included |
| GSM972247 | GSE39582 | IIB/IIC | included |
| GSM972248 | GSE39582 | IIA | included |
| GSM972249 | GSE39582 | I | included |
| GSM972250 | GSE39582 | IIIB | included |
| GSM972251 | GSE39582 | IV | included |
| GSM972252 | GSE39582 | IV | included |
| GSM972253 | GSE39582 | IV | included |
| GSM972254 | GSE39582 | IV | included |
| GSM972255 | GSE39582 | IIA | included |
| GSM972256 | GSE39582 | IV | included |
| GSM972257 | GSE39582 | IIB/IIC | included |
| GSM972258 | GSE39582 | IIB/IIC | included |
| GSM972259 | GSE39582 | IIB/IIC | included |
| GSM972260 | GSE39582 | IV | included |
| GSM972261 | GSE39582 | IIIC | included |
| GSM972262 | GSE39582 | IIB/IIC | included |
| GSM972263 | GSE39582 | IIIA | rejected |
| GSM972264 | GSE39582 | IIA | included |
| GSM972265 | GSE39582 | IIIB | included |
| GSM972266 | GSE39582 | IV | included |
| GSM972267 | GSE39582 | IIA | included |
| GSM972268 | GSE39582 | IIIA | included |
| GSM972269 | GSE39582 | I | included |
| GSM972270 | GSE39582 | NA | rejected |
| GSM972271 | GSE39582 | IIIC | included |
| GSM972272 | GSE39582 | IV | included |
| GSM972273 | GSE39582 | NA | included |
| GSM972274 | GSE39582 | NA | rejected |
| GSM972275 | GSE39582 | NA | rejected |
| GSM972276 | GSE39582 | IIIC | included |
| GSM972277 | GSE39582 | IIB/IIC | included |
| GSM972278 | GSE39582 | I | included |
| GSM972279 | GSE39582 | IIIB | included |
| GSM972280 | GSE39582 | IIB/IIC | included |
| GSM972281 | GSE39582 | I | included |
| GSM972282 | GSE39582 | NA | rejected |
| GSM972283 | GSE39582 | NA | rejected |
| GSM972284 | GSE39582 | IV | included |
| GSM972285 | GSE39582 | I | included |
| GSM972286 | GSE39582 | IIA | included |
| GSM972287 | GSE39582 | IIA | included |
| GSM972288 | GSE39582 | IV | included |
| GSM972289 | GSE39582 | IV | included |
| GSM972290 | GSE39582 | I | included |
| GSM972291 | GSE39582 | IIIC | included |
| GSM972292 | GSE39582 | IIA | included |
| GSM972293 | GSE39582 | I | included |
| GSM972294 | GSE39582 | IIA | included |
| GSM972295 | GSE39582 | IIIC | included |
| GSM972296 | GSE39582 | NA | included |
| GSM972297 | GSE39582 | I | included |
| GSM972298 | GSE39582 | IIA | included |
| GSM972299 | GSE39582 | IV | included |
| GSM972300 | GSE39582 | NA | included |
| GSM972301 | GSE39582 | IIIC | rejected |
| GSM972302 | GSE39582 | IIIC | included |
| GSM972303 | GSE39582 | I | included |
| GSM972304 | GSE39582 | IIA | included |
| GSM972305 | GSE39582 | IIIC | rejected |
| GSM972306 | GSE39582 | IIA | included |
| GSM972307 | GSE39582 | IIA | included |
| GSM972308 | GSE39582 | IIIB | included |
| GSM972309 | GSE39582 | IIA | included |
| GSM972310 | GSE39582 | IIIB | rejected |
| GSM972311 | GSE39582 | IIA | included |
| GSM972312 | GSE39582 | IIA | included |
| GSM972313 | GSE39582 | I | included |
| GSM972314 | GSE39582 | IIIB | included |
| GSM972315 | GSE39582 | I | included |
| GSM972316 | GSE39582 | IIA | included |
| GSM972317 | GSE39582 | IIA | included |
| GSM972318 | GSE39582 | IIA | included |
| GSM972319 | GSE39582 | IIA | included |
| GSM972320 | GSE39582 | IIIA | included |
| GSM972321 | GSE39582 | IIIC | rejected |
| GSM972322 | GSE39582 | IIA | rejected |
| GSM972323 | GSE39582 | IIIB | included |
| GSM972324 | GSE39582 | IIA | included |
| GSM972325 | GSE39582 | IIIB | included |
| GSM972326 | GSE39582 | IIA | included |
| GSM972327 | GSE39582 | IIA | rejected |
| GSM972328 | GSE39582 | IIIC | included |
| GSM972329 | GSE39582 | IIA | rejected |
| GSM972330 | GSE39582 | IIA | included |
| GSM972331 | GSE39582 | IIA | included |
| GSM972332 | GSE39582 | IIA | included |
| GSM972333 | GSE39582 | IIA | included |
| GSM972334 | GSE39582 | IIIC | included |
| GSM972335 | GSE39582 | I | included |
| GSM972336 | GSE39582 | IIIB | included |
| GSM972337 | GSE39582 | IIIB | rejected |
| GSM972338 | GSE39582 | IIA | rejected |
| GSM972339 | GSE39582 | IIIB | included |
| GSM972340 | GSE39582 | IIA | included |
| GSM972341 | GSE39582 | IIIB | included |
| GSM972342 | GSE39582 | I | rejected |
| GSM972343 | GSE39582 | IIA | included |
| GSM972344 | GSE39582 | IIA | included |
| GSM972345 | GSE39582 | IIIC | included |
| GSM972346 | GSE39582 | IIIB | included |
| GSM972347 | GSE39582 | IIA | included |
| GSM972348 | GSE39582 | IIA | included |
| GSM972349 | GSE39582 | IIIC | included |
| GSM972350 | GSE39582 | IIIB | included |
| GSM972351 | GSE39582 | IIB/IIC | rejected |
| GSM972352 | GSE39582 | IIIC | included |
| GSM972353 | GSE39582 | IIB/IIC | included |
| GSM972354 | GSE39582 | IIIC | included |
| GSM972355 | GSE39582 | IIA | rejected |
| GSM972356 | GSE39582 | IIA | included |
| GSM972357 | GSE39582 | IIA | rejected |
| GSM972358 | GSE39582 | IIA | rejected |
| GSM972359 | GSE39582 | IIA | rejected |
| GSM972360 | GSE39582 | IIIB | included |
| GSM972361 | GSE39582 | IIIC | included |
| GSM972362 | GSE39582 | IIIB | rejected |
| GSM972363 | GSE39582 | IIIB | rejected |
| GSM972364 | GSE39582 | IIIB | included |
| GSM972365 | GSE39582 | IIIC | included |
| GSM972366 | GSE39582 | IIIC | rejected |
| GSM972367 | GSE39582 | IIIB | included |
| GSM972368 | GSE39582 | IIIB | included |
| GSM972369 | GSE39582 | IIIC | included |
| GSM972370 | GSE39582 | IIIB | included |
| GSM972371 | GSE39582 | IIIB | included |
| GSM972372 | GSE39582 | IIIC | rejected |
| GSM972373 | GSE39582 | IIIB | included |
| GSM972374 | GSE39582 | IIIA | rejected |
| GSM972375 | GSE39582 | IIIB | included |
| GSM972376 | GSE39582 | IIIC | included |
| GSM972377 | GSE39582 | IIIB | included |
| GSM972378 | GSE39582 | IIIC | included |
| GSM972379 | GSE39582 | IIIA | included |
| GSM972380 | GSE39582 | IIIC | included |
| GSM972381 | GSE39582 | IIA | rejected |
| GSM972382 | GSE39582 | IIA | included |
| GSM972383 | GSE39582 | IIA | rejected |
| GSM972384 | GSE39582 | IIA | included |
| GSM972385 | GSE39582 | IIA | included |
| GSM972386 | GSE39582 | IIA | included |
| GSM972387 | GSE39582 | IIA | included |
| GSM972388 | GSE39582 | IIA | included |
| GSM972389 | GSE39582 | IIB/IIC | included |
| GSM972390 | GSE39582 | IIIB | included |
| GSM972391 | GSE39582 | IIA | included |
| GSM972392 | GSE39582 | IIB/IIC | included |
| GSM972393 | GSE39582 | IIA | included |
| GSM972394 | GSE39582 | IIIB | rejected |
| GSM972395 | GSE39582 | IIIC | included |
| GSM972396 | GSE39582 | IIA | included |
| GSM972397 | GSE39582 | IIA | included |
| GSM972398 | GSE39582 | IIA | included |
| GSM972399 | GSE39582 | IIIC | included |
| GSM972400 | GSE39582 | IV | rejected |
| GSM972401 | GSE39582 | IIA | included |
| GSM972402 | GSE39582 | IV | included |
| GSM972403 | GSE39582 | IIA | included |
| GSM972404 | GSE39582 | IIA | rejected |
| GSM972405 | GSE39582 | IIB/IIC | included |
| GSM972406 | GSE39582 | IIA | included |
| GSM972407 | GSE39582 | IIB/IIC | included |
| GSM972408 | GSE39582 | IIA | included |
| GSM972409 | GSE39582 | IIB/IIC | included |
| GSM972410 | GSE39582 | IIA | included |
| GSM972411 | GSE39582 | IIIA | included |
| GSM972412 | GSE39582 | IIIC | rejected |
| GSM972413 | GSE39582 | IIIB | included |
| GSM972414 | GSE39582 | IIA | included |
| GSM972415 | GSE39582 | IIIB | included |
| GSM972416 | GSE39582 | IIB/IIC | included |
| GSM972417 | GSE39582 | IIA | included |
| GSM972418 | GSE39582 | IIA | included |
| GSM972419 | GSE39582 | IIIC | included |
| GSM972420 | GSE39582 | IIIC | included |
| GSM972421 | GSE39582 | NA | included |
| GSM972422 | GSE39582 | NA | included |
| GSM972423 | GSE39582 | NA | rejected |
| GSM972424 | GSE39582 | IIA | included |
| GSM972425 | GSE39582 | IIIB | included |
| GSM972426 | GSE39582 | IIA | rejected |
| GSM972427 | GSE39582 | IIA | included |
| GSM972428 | GSE39582 | IIIC | included |
| GSM972429 | GSE39582 | IIIC | rejected |
| GSM972430 | GSE39582 | IIA | included |
| GSM972431 | GSE39582 | IIIB | included |
| GSM972432 | GSE39582 | NA | rejected |
| GSM972433 | GSE39582 | NA | included |
| GSM972434 | GSE39582 | NA | included |
| GSM972435 | GSE39582 | NA | included |
| GSM972436 | GSE39582 | NA | included |
| GSM972437 | GSE39582 | IV | included |
| GSM972438 | GSE39582 | IV | included |
| GSM972439 | GSE39582 | IV | rejected |
| GSM972440 | GSE39582 | IIA | included |
| GSM972441 | GSE39582 | IV | rejected |
| GSM972442 | GSE39582 | IV | rejected |
| GSM972443 | GSE39582 | IIA | included |
| GSM972444 | GSE39582 | NA | rejected |
| GSM972445 | GSE39582 | NA | rejected |
| GSM972446 | GSE39582 | IIIB | rejected |
| GSM972447 | GSE39582 | IIA | rejected |
| GSM972448 | GSE39582 | IIA | included |
| GSM972449 | GSE39582 | IIIC | rejected |
| GSM972450 | GSE39582 | IIA | included |
| GSM972451 | GSE39582 | IIB/IIC | included |
| GSM972452 | GSE39582 | IIIC | included |
| GSM972453 | GSE39582 | IIIB | included |
| GSM972454 | GSE39582 | I | rejected |
| GSM972455 | GSE39582 | IIIB | included |
| GSM972456 | GSE39582 | IIA | included |
| GSM972457 | GSE39582 | IIIB | included |
| GSM972458 | GSE39582 | IIA | included |
| GSM972459 | GSE39582 | NA | rejected |
| GSM972460 | GSE39582 | IIA | included |
| GSM972461 | GSE39582 | IIA | included |
| GSM972462 | GSE39582 | I | included |
| GSM972463 | GSE39582 | IIIC | included |
| GSM972464 | GSE39582 | I | included |
| GSM972465 | GSE39582 | IIA | included |
| GSM972466 | GSE39582 | IIIB | included |
| GSM972467 | GSE39582 | IIIC | included |
| GSM972468 | GSE39582 | IIA | included |
| GSM972469 | GSE39582 | I | included |
| GSM972470 | GSE39582 | IIA | included |
| GSM972471 | GSE39582 | I | included |
| GSM972472 | GSE39582 | IIB/IIC | included |
| GSM972473 | GSE39582 | IIA | rejected |
| GSM972474 | GSE39582 | IIIB | rejected |
| GSM972475 | GSE39582 | IIA | included |
| GSM972476 | GSE39582 | IIIC | included |
| GSM972477 | GSE39582 | IIA | included |
| GSM972478 | GSE39582 | IIA | rejected |
| GSM972479 | GSE39582 | IIIC | included |
| GSM972480 | GSE39582 | IIA | included |
| GSM972481 | GSE39582 | IIIC | included |
| GSM972482 | GSE39582 | IIA | included |
| GSM972483 | GSE39582 | IIA | included |
| GSM972484 | GSE39582 | IIA | included |
| GSM972485 | GSE39582 | IIA | included |
| GSM972486 | GSE39582 | IIA | included |
| GSM972487 | GSE39582 | IIA | included |
| GSM972488 | GSE39582 | IIA | included |
| GSM972489 | GSE39582 | IIA | included |
| GSM972490 | GSE39582 | IIA | included |
| GSM972491 | GSE39582 | IIA | included |
| GSM972492 | GSE39582 | I | included |
| GSM972493 | GSE39582 | IIA | included |
| GSM972494 | GSE39582 | NA | rejected |
| GSM972495 | GSE39582 | IIIB | included |
| GSM972496 | GSE39582 | I | included |
| GSM972497 | GSE39582 | IIIA | included |
| GSM972498 | GSE39582 | IIIB | included |
| GSM972499 | GSE39582 | IIIC | included |
| GSM972500 | GSE39582 | IIA | rejected |
| GSM972501 | GSE39582 | IIB/IIC | rejected |
| GSM972502 | GSE39582 | IIA | included |
| GSM972503 | GSE39582 | IIA | rejected |
| GSM972504 | GSE39582 | IIA | rejected |
| GSM972505 | GSE39582 | IIA | rejected |
| GSM972506 | GSE39582 | IIA | rejected |
| GSM972507 | GSE39582 | IIIB | included |
| GSM972508 | GSE39582 | I | rejected |
| GSM972509 | GSE39582 | I | included |
| GSM972510 | GSE39582 | IIA | included |
| GSM972511 | GSE39582 | IIIC | included |
| GSM972512 | GSE39582 | IIIC | included |
| GSM972513 | GSE39582 | IIIB | included |
| GSM972514 | GSE39582 | IIIA | included |
| GSM972515 | GSE39582 | IIA | included |
| GSM972516 | GSE39582 | NA | rejected |
| GSM972517 | GSE39582 | IIA | included |
| GSM972518 | GSE39582 | IIIC | rejected |
| GSM972519 | GSE39582 | IIA | rejected |
| GSM972520 | GSE39582 | IIIB | rejected |
| GSM972521 | GSE39582 | IIA | included |
| GSM972522 | GSE39582 | IIA | rejected |
